# 20-Hydroxyecdysone counteracts insulin to promote programmed cell death by modifying phosphoglycerate kinase 1

**DOI:** 10.1186/s12915-023-01621-2

**Published:** 2023-05-24

**Authors:** Xin-Le Kang, Yan-Xue Li, Du-Juan Dong, Jin-Xing Wang, Xiao-Fan Zhao

**Affiliations:** Shandong Provincial Key Laboratory of Animal Cells and Developmental Biology, School of Life Sciences, Shandong University, Qingdao, 266237 China

**Keywords:** PGK1 posttranslational regulation, Insulin, 20-Hydroxyecdysone, Glycolysis, Programmed cell death, Metamorphosis

## Abstract

**Background:**

The regulation of glycolysis and autophagy during feeding and metamorphosis in holometabolous insects is a complex process that is not yet fully understood. Insulin regulates glycolysis during the larval feeding stage, allowing the insects to grow and live. However, during metamorphosis, 20-hydroxyecdysone (20E) takes over and regulates programmed cell death (PCD) in larval tissues, leading to degradation and ultimately enabling the insects to transform into adults. The precise mechanism through which these seemingly contradictory processes are coordinated remains unclear and requires further research. To understand the coordination of glycolysis and autophagy during development, we focused our investigation on the role of 20E and insulin in the regulation of phosphoglycerate kinase 1 (PGK1). We examined the glycolytic substrates and products, PGK1 glycolytic activity, and the posttranslational modification of PGK1 during the development of *Helicoverpa armigera* from feeding to metamorphosis.

**Results:**

Our findings suggest that the coordination of glycolysis and autophagy during holometabolous insect development is regulated by a balance between 20E and insulin signaling pathways. Glycolysis and PGK1 expression levels were decreased during metamorphosis under the regulation of 20E. Insulin promoted glycolysis and cell proliferation via PGK1 phosphorylation, while 20E dephosphorylated PGK1 via phosphatase and tensin homolog (PTEN) to repress glycolysis. The phosphorylation of PGK1 at Y194 by insulin and its subsequent promotion of glycolysis and cell proliferation were important for tissue growth and differentiation during the feeding stage. However, during metamorphosis, the acetylation of PGK1 by 20E was key in initiating PCD. Knockdown of phosphorylated PGK1 by RNA interference (RNAi) at the feeding stage led to glycolysis suppression and small pupae. Insulin via histone deacetylase 3 (HDAC3) deacetylated PGK1, whereas 20E via acetyltransferase arrest-defective protein 1 (ARD1) induced PGK1 acetylation at K386 to stimulate PCD. Knockdown of acetylated-PGK1 by RNAi at the metamorphic stages led to PCD repression and delayed pupation.

**Conclusions:**

The posttranslational modification of PGK1 determines its functions in cell proliferation and PCD. Insulin and 20E counteractively regulate PGK1 phosphorylation and acetylation to give it dual functions in cell proliferation and PCD.

**Supplementary Information:**

The online version contains supplementary material available at 10.1186/s12915-023-01621-2.

## Background

Holometabolous insects feed and grow at the larval stage and stop feeding and pupate during metamorphosis. Metamorphosis is a time-controlled life transition event that relies solely on the accumulation of energetic macromolecules for energy supply during the larval stage. Metabolism must be reprogrammed for the developmental transition to meet the energy demands of an organism’s life cycle. Glucose metabolism is crucial for energy homeostasis during the larval-pupal transition and is associated with fat body re-architecture in *Helicoverpa armigera* [1]. Glucose can directly generate pyruvate, lactate, and adenosine triphosphate (ATP) in vivo through glycolysis. Glycolysis decreased in the midgut of *Spodoptera litura* prepupae during metamorphosis to save energy. However, pupae upregulated glycolysis to provide intermediates for adult tissues reconstruction [2]. *Bombyx* insulin-like peptide injection into *Bombyx mori* larvae have been shown to increase the glycolytic activity of tissues [3]. Most genes encoding glycolytic enzymes are down-regulated during metamorphosis initiation in *Drosophila melanogaster* [4]. Glucose metabolism is reprogrammed by 20E, accumulating glucose during the larval-pupal transition [1, 5]. However, the molecular mechanisms that regulate glucose homeostasis during metamorphosis remain largely unknown.

During feeding stages, insect insulin-like peptides (ILPs) function similarly to human insulin through insulin/insulin-like growth factor (IGF) signaling (IIS) [6] to promote cell proliferation and body growth [7]. However, the insulin pathway is repressed and the programmed cell death (PCD), including autophagy [8] and apoptosis [9], is promoted by the increased concentrations of 20-hydroxyecdysone (20E) during metamorphosis [5, 10]. Glucose-derived substances and energy for the proliferation and differentiation of imaginal discs during metamorphosis originate from autophagy in the larval tissues’ lysis [11–13]. During *D. melanogaster* and *B. mori* metamorphosis, 20E induces autophagy [14–16] and apoptosis [12, 17] in the fat body. Despite extensive research on 20E’s regulatory roles in metamorphosis, the mechanism of metamorphosis achieved by the temporal regulation of glucose metabolism and PCD by steroid hormones and ILPs is poorly understood.

Phosphoglycerate kinase 1 (PGK1) catalyzes the reversible reaction, converting 1,3-bisphosphoglycerate (1,3-BPG) and ADP to 3-phosphoglycerate (3-PG) and ATP, thereby playing a vital role in coordinating energy conservation with biosynthesis [18]. *PGK1* expression in the fat body is high during the feeding larval stages and low in the prepupae stages [19]. High levels of PGK1 promote glycolysis for energy conservation and the generation of biosynthetic intermediates in many types of human cancer, including breast cancer [20] and metastatic gastric cancer [21]. Post-translational regulation is imperative for PGK1 functioning. Specifically, PGK1 O-linked N-acetylglucosamine (O-GlcNAc) at T255 activates PGK1 activity to enhance lactate production [22]. Furthermore, insulin promotes PGK1 K220 deacetylation to stimulate PGK1 glycolytic activity in HEK293T cells [18]. Aside from its glycolytic activity, acetylated PGK1 acts as a protein kinase to induce autophagy by phosphorylating Beclin1 during brain tumorigenesis [23]. Phosphorylated PGK1 promotes pyruvate dehydrogenase kinase 1 T338 phosphorylation in mitochondria, thereby regulating mitochondrial metabolism during brain tumorigenesis [24]. Although PGK1 plays diverse roles in different cellular processes via different post-translational modifications, the coordination of metabolic homeostasis and PCD through hormonal signaling mediated-PGK1 posttranslational regulations for the development of animal body remodeling, particularly the larval-pupal transition, remains elusive.

In this study, the regulatory mechanism of 20E antagonizing insulin was evaluated by examining the post-translational modification of PGK1 in the prevalent lepidopteron pest, *H. armigera*. During insect metamorphosis, elevated 20E antagonizes insulin-mediated phosphorylation of PGK1, which promotes glycolytic activity, as well as induces PGK1 acetylation, which initiates autophagy. This report reveals multiple functions of the glycolytic enzyme PGK1 with different posttranslational modifications during insect metamorphosis. Phosphorylated PGK1 plays a crucial role in glycolysis during feeding and growth stages, and PGK1 acetylation plays a major role in autophagy and apoptosis during metamorphosis. This transition between posttranslational modification and function is closely related to insulin and 20E regulation. Our findings lead to the hypothesis that PGK1 posttranslational regulation not only defines the reprogrammed regulation of glucose metabolism but also facilitates pupation by inducing PCD.

## Results

### Glycolytic activity and *PGK1* expression are downregulated by 20E

**Fig. 1:**
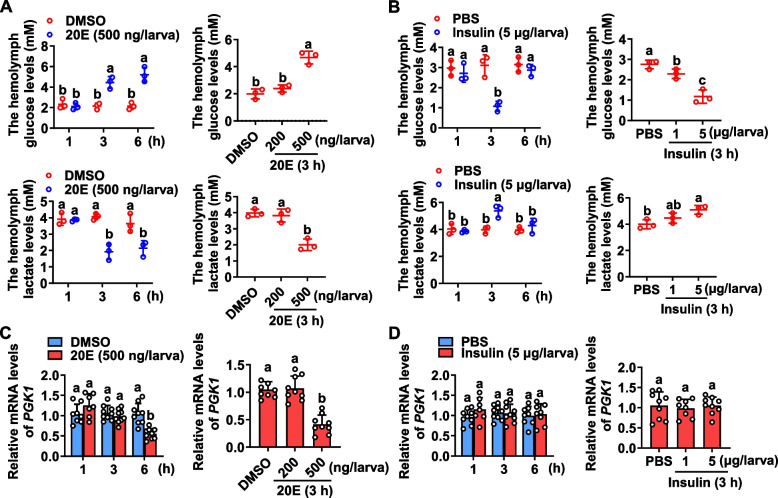
High titers of 20E oppose the insulin effect in regulating glycolysis and repressing *PGK1* expression. **A** Glucose and lactate levels in hemolymph after 20E injection over time and dosage. *p* < 0.05, *n* = 3. **B** Glucose and lactate levels in hemolymph after insulin injection across time and dosages. *p* < 0.05, *n* = 3. **C** qRT-PCR analysis of the downregulation of *PGK1* in the larval fat body over time and dosage in response to 20E. *p* < 0.05, *n* = 8 or 9. **D** qRT-PCR detection of *PGK1* expression in the larval fat body based on insulin injection over time and dosage. *p* < 0.05, *n* = 8 or 9. DMSO or PBS was used as a solvent control for same time. 500 ng/larva of 20E or 5 μg/larva of insulin was injected into sixth-instar larvae at 6 h. Bars indicate means ± SD for more than three independent experiments with five larvae per replicate. Statistically significant differences were calculated using one-way analysis of variance (ANOVA, *p* < 0.05) tests

To investigate the glycolytic activity during development, the levels of glycolytic substrate glucose and product lactate were measured. Glucose concentrations increased from the feeding stages (the sixth-instar from 24 to 72 h) to the wandering stages (the sixth-instar from 96 to 120 h) and were high during metamorphosis, whereas lactate levels decreased from the feeding stages to the wandering stages (Additional file 1: Fig. S1A). In addition, 20E treatment increased larval hemolymph glucose levels, while decreased hemolymph lactate levels in time- and dose-dependent manners compared with dimethyl sulfoxide (DMSO) controls (Fig. 1A). Larval hemolymph glucose levels decreased, while hemolymph lactate levels increased in time- and dose-dependent manners after insulin injection compared with phosphate-buffered saline (PBS) controls (Fig. 1B). These data indicated that insulin increases glycolytic activity, whereas 20E decreases glycolytic activity during insect development.

PGK1, the first ATP-generating enzyme in the glycolytic pathway, is crucial for coordinating energy production with biosynthesis [18]. To investigate the function of PGK1 in *H. armigera* larval development, expression profiles of *PGK1* during development were examined. *PGK1* mRNA levels in the fat body increased during the feeding stages of the fifth-instar (5F), as well as during the sixth-instar from 24 to 72 h, and then decreased during the sixth-instar’s wandering stage (96 h to 120 h) and were low during the pupal stages (Additional file 1: Fig. S1B). *PGK1* expression in the fat body decreased in a time- and dose-dependent manner after 20E injection into sixth-instar larvae at 6 h (Fig. 1C). However, *PGK1 * expression did not change in response to insulin induction (Fig. 1D). Thus, the expression of *PGK1* is repressed by high levels of 20E.

### Insulin and 20E counteractively regulate PGK1 phosphorylation and acetylation

**Fig. 2:**
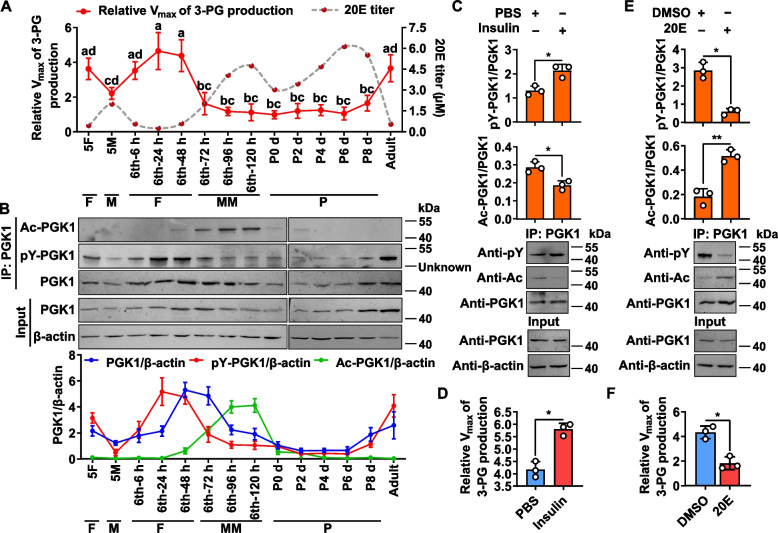
Insulin promotes PGK1 phosphorylation and represses PGK1 acetylation, but 20E does the opposite in vivo. **A** Vmax of 3-PG production in the fat body and 20E titers (according to [25]) from 5F to adult stages. *p* < 0.05, *n* = 3. **B** Levels of PGK1, PGK1 phosphorylation, and PGK1 acetylation in the fat body from 5F to adult stages. PGK1 proteins from the fat body were purified with CNBr-activated Sepharose 4B and an anti-PGK1 monoclonal antibody, followed by in vitro detection. 5F, fifth-instar feeding larvae; 5 M, fifth-instar molting larvae; 6th-6 h to 6th-120 h represent sixth-instar larvae at the corresponding hours. P-0 d to P-8 d denote 0- to 8-day-old pupae. F, feeding; M, molting; MM, metamorphic molting; P, pupae. An additional band at about 40 kDa appeared in pY immunoprecipitation indicated as Unknown. *n* = 3. **C** Insulin increased PGK1 phosphorylation but decreased its acetylation. Sixth-instar larvae at 48 h were injected with 5 μg insulin/larva for 3 h. *p < 0.05, *n* = 3. **D** Insulin enhanced PGK1 glycolytic activity based on the detection of Vmax for 3-PG production in **C**. *p < 0.05, *n* = 3. **E** 20E reduced PGK1 phosphorylation but increased its acetylation. Sixth-instar larvae at 48 h were injected with 500 ng 20E/larva for 3 h. *p < 0.05, **p < 0.01, *n* = 3. **F** As measured by the Vmax for 3-PG production, 20E decreased PGK1 glycolytic activity in **E**. *p < 0.05, *n* = 3. PGK1 proteins from the fat body were purified by CNBr-activated Sepharose 4B with anti-PGK1 monoclonal antibody before western blot analysis and the detection of Vmax for 3-PG production. 12.5% SDS-PAGE gels were used for western blot and β-actin was used for protein quantity control. Data represent means ± SD for three independent experiments with five larvae per replicate. Statistically significant differences were calculated using two-tailed Student’s *t* tests (*: *p* < 0.05, **p < 0.01) or one-way analysis of variance (ANOVA, *p* < 0.05) tests. pY, phosphorylated tyrosine. Ac, acetylated lysine

The amino acid sequence of *H. armigera* PGK1 is conserved highly in insects and *Homo sapiens* (Additional file 1: Fig. S2). PGK1 from the fat body was purified with an anti-PGK1 antibody to determine the profiles of PGK1-catalyzed reaction toward glycolysis. As measured by the maximum reaction rate (Vmax) of 3-PG production, PGK1 glycolytic activity increased at feeding stages but decreased at metamorphic stages. This is contrary to the trend in 20E titers (Fig. 2A). The post-translational modification (PTM) of PGK1 was investigated to assess its function in glycolysis during feeding and metamorphosis. The phosphorylated PGK1 of the fat body was increased at the sixth-instar feeding stages and decreased at the metamorphic stages based on the use of the anti-pY (phosphated tyrosine) antibody (Fig. 2B). At the metamorphic stages, PGK1 expression levels were also decreased (Fig. 2B). Notably, the use of anti-Ac (acetylated lysine) antibodies revealed that PGK1 was acetylated during the metamorphic stage in the fat body (Fig. 2B). These results indicated that PGK1 is phosphorylated during feeding and acetylated during metamorphosis.

Insulin plays a considerable role in feeding and growth [26], while 20E plays a major role in metamorphosis [27, 28]. Therefore, hormonal regulation of PGK1 PTM was evaluated to determine the function of PGK1 during feeding and metamorphosis. PGK1 protein was isolated from the fat body using an anti-PGK1 antibody after injection of insulin or 20E into sixth-instar 48 h larvae for 3 h. Insulin induced PGK1 phosphorylation and inhibited PGK1 acetylation (Fig. 2C), which promoted the PGK1-catalyzed glycolysis reaction (Fig. 2D). Moreover, 20E inhibited PGK1 phosphorylation but promoted PGK1 acetylation (Fig. 2E), which repressed the PGK1-catalyzed reaction toward glycolysis (Fig. 2F). These results suggested that PGK1 phosphorylation and acetylation are counteractively regulated by insulin and 20E.

### PGK1 Y194 phosphorylation promotes PGK1 glycolytic activity and 20E induces dephosphorylation of PGK1 via phosphatase and tensin homolog (PTEN)

**Fig. 3:**
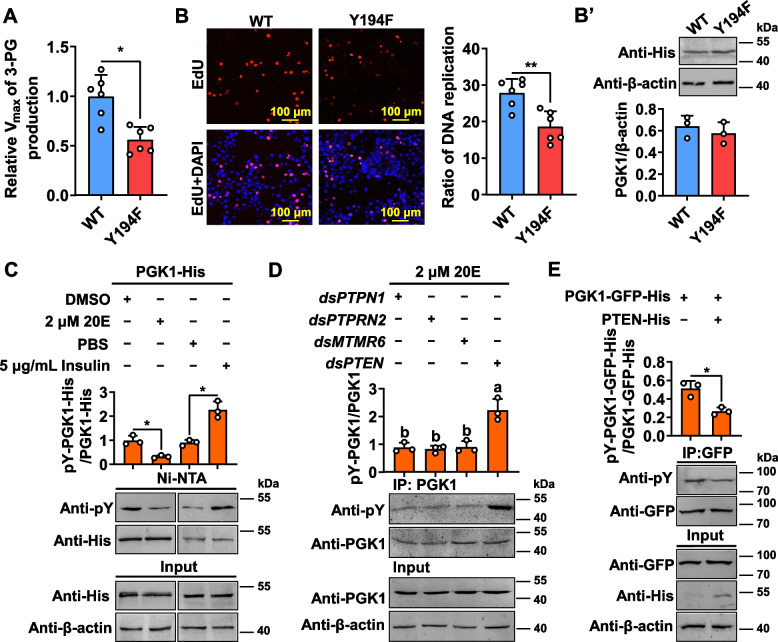
Phosphorylation of PGK1 at Y194 enhances PGK1 glycolytic activity and PTEN dephosphorylates PGK1. **A** Phosphorylation of PGK1 at Y194 promotes glycolysis based on the detection of Vmax of 3-PG production in vitro. Wild type PGK1 (WT) and mutant PGK1 (Y194F) were overexpressed in HaEpi cells for 48 h, respectively, and then incubated with insulin (5 μg/mL) for 3 h. PGK1 proteins were purified by Ni–NTA agarose beads to detect the Vmax of 3-PG production. *p < 0.05, *n* = 6. **B** and **B’** PGK1 Y194 phosphorylation promotes cell proliferation. HaEpi cells were overexpressed with PGK1 WT or PGK1 Y194F, and incubated with 5 μg/mL insulin for 3 h, followed by proliferation signal detection using an EdU kit (**B**). The ratio of proliferation cells (red) to total cells (blue, nuclei stained with DAPI) in the field of view was assessed, and data were expressed as mean ± SD from 100 × 6 cells for statistical analysis. **p < 0.01, *n* = 6. Western blot validation of PGK1 WT and PGK1 Y194F mutant overexpression efficiency in HaEpi cells (**B’**). *n* = 3. **C** PGK1 is dephosphorylated by 20E and phosphorylated by insulin. HaEpi cells overexpressing PGK1-His were treated with 20E or insulin for 3 h. PBS or DMSO were used as controls. PGK1 proteins were purified with Ni–NTA agarose beads. *p < 0.05, *n* = 3. **D** 20E dephosphorylates PGK1 via *PTEN*. dsRNA knockdown (500 ng/larva administered in the sixth-instar at 6 h, three times over 24 h intervals), followed by incubation with 2 μM 20E for 3 h. PGK1 proteins from the fat body were purified by CNBr-activated Sepharose 4B with the anti-PGK1 monoclonal antibody. *p* < 0.05, *n* = 3. **E** PTEN overexpression decreased the phosphorylation levels of PGK1. HaEpi cells overexpressing PGK1-GFP-His were transfected with PTEN-His or not, followed by incubation with 2 μM 20E for 3 h. Anti-GFP antibody immunoprecipitated PGK1-GFP-His, followed by detection using the antibody against phosphorylated tyrosine. *p < 0.05, *n* = 3. Data represent means ± SD for more than three independent experiments with one six-well cell culture plates or five larvae per replicate. Statistically significant differences were calculated using two-tailed Student’s *t* tests (*: *p* < 0.05 and **: *p* < 0.01) or one-way analysis of variance (ANOVA, *p* < 0.05) tests. pY, the antibody against phosphorylated tyrosine

Liquid chromatography/tandem mass spectrometry (LC–MS/MS) analysis was used to identify the phosphorylation site of the PGK1 protein purified from the fat body of larvae in the feeding stage. As shown in Fig. S3 (Additional file 1), PGK1 was phosphorylated at Tyr194. This phosphorylation site of PGK1 and its consequence was identified by PGK1 mutation. Compared to the PGK1 WT (PGK1-His), the PGK1 Y194F mutant (phosphorylated tyrosine was replaced by phenylalanine) protein had a lower Vmax of 3-PG production (Fig. 3A). 5-Ethynyl-2′-deoxyuridine (EdU) assay was used to determine the effect of insulin-induced tyrosine phosphorylation of PGK1 on cell proliferation. By comparison to WT-overexpressing *H. armigera* epidermal cell line (HaEpi) cells, Y194F-overexpressing HaEpi cells displayed a low proliferative EdU signal (Fig. 3B and B’). These results suggested that PGK1 plays a key role in glycolysis and cellular proliferation through the phosphorylation of Y194.

PGK1 phosphorylation levels were measured in HaEpi cells overexpressing PGK1-His with 20E and insulin treatment. In contrast to the negative control, immunoblotting showed that PGK1 phosphorylation levels were significantly reduced in 20E treatment but increased following insulin stimulation (Fig. 3C). It has been demonstrated that *PTEN*, as well as three protein-tyrosine phosphatase genes, *PTPN1* (XP_021192174.1), *MTMR6* (XP_021195240.1), and *PTPRN2* (XP_021185000.1), are highly expressed during metamorphosis in *H. armigera*, and upon 20E induction, the expression of these phosphatases increased [5, 29], suggesting that they might be involved in dephosphorylation of PGK1. Thus, dsRNA was used to screen the phosphatases dephosphorylating PGK1 in *H. armigera* (Additional file 1: Fig. S4A). PGK1 phosphorylation was maintained by dsPTEN, but not by dsPTPN1, dsPTPRN2, or dsMTMR6 under 20E induction (Fig. 3D). In contrast, PGK1 phosphorylation was suppressed in HaEpi overexpressing PTEN-His (Fig. 3E). These data indicated that 20E induces dephosphorylation of PGK1 via PTEN.

### PGK1 phosphorylation is necessary for glycolytic activity and larval growth

**Fig. 4:**
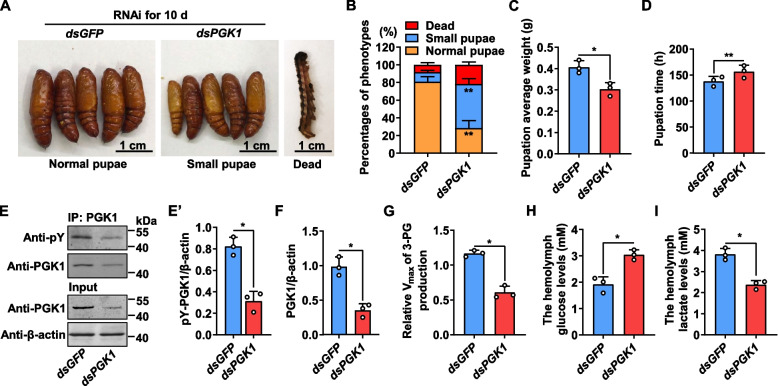
*PGK1* knockdown in the fifth-instar larvae decreased glycolysis and led to small pupa. **A** Phenotypes after injection of dsRNA from 5F to 6th-6 h (500 ng/larva administered in the fifth-instar feeding larvae, three times over 24 h intervals). Images were obtained after RNAi exposure for 10 days. dsGFP was used as a negative control. RNAi was performed on three repeats and 30 larvae for each repeat. **B** Percentages of phenotypes in **A**. **p < 0.01, *n* = 3. **C** The average body weight of pupa after RNAi exposure in **A**. *p < 0.05, *n* = 3. **D** The pupation time from 6th-6 h to pupa 0 d after *PGK1* knockdown in **A**. **p < 0.01, *n* = 3. **E** and **E’** PGK1 phosphorylation levels in dsGFP and dsPGK1 fat body after the third dsRNA injection. PGK1 proteins from the fat body were purified by CNBr-activated Sepharose 4B with an anti-PGK1 monoclonal antibody. *p < 0.05, *n* = 3. **F** Efficiency analysis of *PGK1* knockdown at protein levels from the input of **E**. *p < 0.05, *n* = 3. **G** PGK1 glycolytic activity decreased based on the detection of Vmax for 3-PG production in vitro from **E**. PGK1 proteins from the fat body were purified by CNBr-activated Sepharose 4B with an anti-PGK1 monoclonal antibody, followed by in vitro detection. *p < 0.05, *n* = 3. **H** and **I** The levels of glucose and lactate in *H. armigera* hemolymph after the third dsRNA injection. *p < 0.05, *n* = 3. Data represent means ± SD for three independent experiments with five larvae per replicate. Statistically significant differences were calculated using two-tailed Student’s *t* tests (*p < 0.05 and **p < 0.01). pY, the antibody against phosphorylated tyrosine

To determine the role of PGK1 in larval growth, dsPGK1 was injected into the fifth-instar larvae. By comparison to the dsGFP injection control, phenotype analysis showed that dsPGK1 knockdown resulted in 40% small pupae and decreased pupal weights on average (Fig. 4A–C). Meanwhile, the pupation time was delayed for 18 h after *PGK1* knockdown (Fig. 4D). Western blot analysis showed a decrease in phosphorylated PGK1 and total PGK1 after *PGK1* knockdown when PGK1 was confirmed as knocked down based on its protein levels (Fig. 4E, F). PGK1-catalyzed glycolysis in the fat body was reduced after *PGK1* knockdown (Fig. 4G). *PGK1* knockdown also increased larval hemolymph glucose and decreased larval hemolymph lactate (Fig. 4H and I). Based on these results, it appeared that glycolytic activity and larval growth are dependent on the phosphorylated-PGK1.

### 20E induces PGK1 acetylation and insulin induces PGK1 deacetylation

**Fig. 5:**
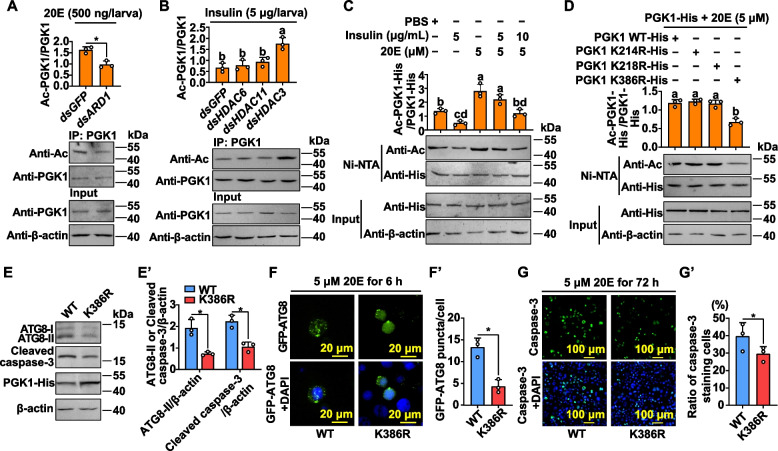
High 20E titer induces PGK1 acetylation to initiate autophagy and apoptosis. **A** High 20E titer induces PGK1 acetylation via *ARD1*. dsARD1 knockdown (500 ng/larva administered in the sixth-instar at 6 h, three times over 24 h intervals) and then injected with 500 ng/larva 20E for 3 h. PGK1 proteins from the fat body were purified by CNBr-activated Sepharose 4B with the anti-PGK1 monoclonal antibody. *p < 0.05, *n* = 3. **B** Insulin stimulates PGK1 deacetylation through *HDAC3*. dsRNA knockdown (500 ng/larva administered in the sixth-instar at 6 h, three times over 24 h intervals) and then injected with 5 μg/larva insulin for 3 h. PGK1 proteins from the fat body were purified by CNBr-activated Sepharose 4B with the anti-PGK1 monoclonal antibody. *p* < 0.05, *n* = 3. **C** High 20E titer facilitates but insulin suppresses PGK1 acetylation, and high concentration of insulin represses 20E-induced PGK1 acetylation. PGK1-His were overexpressed in HaEpi cells, followed by treatment with insulin or 20E for 3 h. PGK1 proteins were purified by Ni–NTA His bound beads. *p* < 0.05, *n* = 3. **D** PGK1 WT-His and its mutants (K214R, K218R, and K386R) were overexpressed in HaEpi cells for 48 h and then treated with 5 μM 20E for 3 h. PGK1 proteins were purified by Ni–NTA His bound beads. *p* < 0.05, *n* = 3. **E** and **E’** Levels of ATG8-II and cleaved-caspase-3 in the PGK1 WT and PGK1 K386R mutant. The PGK1 WT and PGK1 K386R mutant were transfected into HaEpi cells for 48 h and then treated with 5 μM 20E for 6 h. Western blot analysis with anti-ATG8 and anti-caspase-3 antibodies. *p < 0.05, *n* = 3. **F** and **F’** GFP-ATG8 was transiently expressed in HaEpi cells. Representative images of GFP-ATG8 puncta are shown. *p < 0.05, *n* = 3. **G** and **G’** Apoptosis signals were examined via caspase-3 activity using a caspase-3 activity detection kit. *p < 0.05, *n* = 3. Blue: nucleus stained with DAPI. The data represent means ± SD for three independent experiments with one six-well cell culture plates or five larvae per replicate. Statistically significant differences were calculated using two-tailed Student’s *t* tests (*p < 0.05). Different letters indicate statistically significant differences (*p* < 0.05) based on one-way ANOVA tests. Ac, antibodies against pan anti-acetyl lysine

Acetyltransferase arrest-defective protein 1 (ARD1) acetylates PGK1 at K388 in an mTOR-inhibition-dependent manner and the acetylation site K388 is conserved [23]. dsARD1 was injected into the sixth-instar 6 h larvae to investigate whether *ARD1* (XP_021192849.1) was responsible for PGK1 acetylation. The knockdown efficiency of dsARD1 was determined by qRT-PCR (Additional file 1: Fig. S4B). The knockdown of *ARD1* in *H. armigera* plus incubation with 20E led to a decrease in PGK1 acetylation levels without affecting the total PGK1 protein (Fig. 5A). In addition, *ARD1* expression was increased under 20E induction (Additional file 1: Fig. S5A). These results indicated that 20E induces PGK1 acetylation via *ARD1*.

To identify the histone deacetylase (HDAC) responsible for PGK1 deacetylation under insulin regulation, dsHDAC was injected into the sixth-instar larvae, respectively. The knockdown efficiency of each dsHDAC was determined by qRT-PCR (Additional file 1: Fig. S4C). There was a diminished effect of insulin on the reduction of PGK1 acetylation levels in the fat body when *HDAC3* (XP_021195587.1) was knocked down, but neither *HDAC6* (XP_021185594.1) nor *HDAC11* (XP_021187443.1) was affected (Fig. 5B). Furthermore, *HDAC3* expression increased by insulin treatment (Additional file 1: Fig. S5B). These results suggested that insulin promotes PGK1 deacetylation by *HDAC3*.

The role of 20E and insulin in PGK1 acetylation was studied in HaEpi cells that were overexpressing PGK1-His. Western blot with anti-Ac antibody demonstrated that acetylated PGK1 increased by 20E induction, but decreased by insulin treatment in HaEpi cells (Fig. 5C), indicating that high 20E titers stimulated but insulin suppressed PGK1 acetylation. In addition, the acetylation of PGK1 was repressed by higher insulin level (10 μg/mL) treatment in the case of high 20E titers (5 μM) incubation (Fig. 5C), suggesting that a high concentration of insulin could repress PGK1 acetylation induced by high 20E titer.

### PGK1 K386 acetylation promotes autophagy and apoptosis

To determine which lysine residue(s) of PGK1 are acetylated by 20E induction, three putative acetylated lysine residues were mutated into arginine (R), followed by assaying acetylation levels. PGK1 K386R-His, but not PGK1 K214R-His or PGK1 K218R-His, substantially reduced PGK1 acetylation levels (Fig. 5D), indicating the K386 was acetylated in PGK1.

PGK1 acetylation was further evaluated to assess its role in autophagy and apoptosis. Autophagy and apoptosis between PGK1 WT and PGK1 K386R were evaluated to assess the functional role of PGK1 K386 acetylation. In comparison to PGK1 WT, the PGK1 K386R mutant repressed the conversion of ATG8-I to ATG8-II and the generation of cleaved caspase-3 (Fig. 5E and E’). Inhibition of lysosomal activity in HaEpi cells overexpressed with PGK1 WT by chloroquine (CQ) increased ATG8-II expression, but ATG8-II expression was no change in HaEpi cells overexpressed with PGK1 K386R mutant incubated with CQ or not (Additional file 1: Fig. S5C). Further, 20E-induced autophagosome formation was repressed in the K386R mutant, as reflected by the presence of ATG8 puncta in HaEpi cells (Fig. 5F and F’). Moreover, PGK1 K386R mutant expression largely inhibited 20E-induced HaEpi cells’ apoptosis compared with PGK1 WT expression (Fig. 5G and G’). However, the mutant K386R did not affect PGK1 phosphorylation (Additional file 1: Fig. S5D) and PGK1 glycolytic ability (Additional file 1: Fig. S5E), indicating that PGK1 acetylation-initiated autophagy is independent of PGK1 phosphorylation. Consequently, 20E induces PGK1 K386 acetylation, which in turn promotes cell autophagy and apoptosis.

### PGK1 acetylation is vital for autophagy and apoptosis during insect metamorphosis

Compared to sixth-instar larvae at 48 h, sixth-instar larvae at 96 h have a constricted body length and a degraded fat body (Additional file 1: Fig. S6A, B), indicating the 6th-96 h larvae are in the wandering stage. To assess the function of PGK1 in autophagy and apoptosis during insect metamorphosis, the development of the fat body from the sixth-instar at 48 h (feeding stage) and the sixth-instar at 96 h (metamorphic stage) was monitored. The fat body was degraded, with caspase-3 localized primarily in the nuclei from the sixth-instar at 96 h compared with the sixth-instar at 48 h (Additional file 1: Fig. S6B, B’), suggesting that apoptosis occurred in the sixth-instar at 96 h. Transmission electron microscopy (TEM) analysis revealed that autophagosomes, autolysosomes, and apoptotic nuclei were more prevalent in the sixth-instar larvae at 96 h than in the sixth-instar at 48 h (Additional file 1: Fig. S6C, C’). Additionally, western blot further confirmed that caspase-3 cleavage and the conversion of ATG8-I to ATG8-II increased in the sixth-instar at 96 h in comparison with the sixth-instar at 48 h (Additional file 1: Fig. S6D and D’). These data suggested that autophagy and apoptosis occur during metamorphosis.

**Fig. 6:**
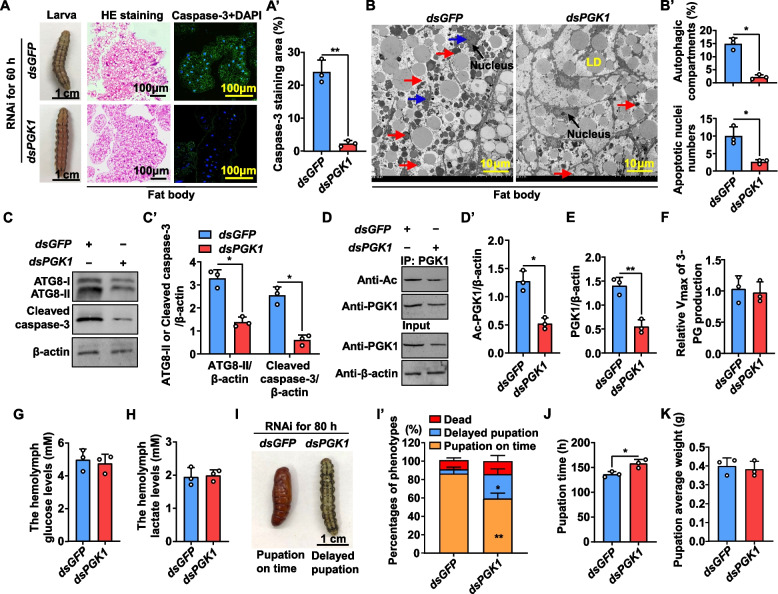
*PGK1* knockdown at the wandering stage delays metamorphosis. **A** Morphology, HE staining, and caspase-3 location in the fat body after dsGFP and dsPGK1 knockdown. *PGK1* knockdown (500 ng/larva administered in the sixth-instar at 72 h, three times over 24 h intervals). Images were obtained after the first RNAi exposure for 60 h. dsGFP was used as a negative control. Rabbit polyclonal antibodies against caspase-3 were used as the primary antibody. Green fluorescence indicates caspase-3 presence. Nuclei were stained with DAPI (blue). **A’** Quantification of fluorescent caspase-3 in **A**. **p < 0.01, *n* = 3. **B** TEM analysis of the fat body from **A**. LD indicates lipid droplets. Red arrows indicate autolysosomes or autophagosomes. Blue arrows represent the apoptotic nuclei. **B’** Quantification of autophagosomes, autolysosomes, and apoptotic nucleus in **B**. *p < 0.05, *n* = 3. **C** and **C’** The levels of ATG8-II and cleaved-caspase-3 in the fat body based on **A**. Western blot detection using anti-ATG8 and anti-caspase-3 antibodies. *p < 0.05, *n* = 3. **D** and **D’** PGK1 acetylation levels in dsGFP and dsPGK1 fat body from **A**. PGK1 proteins from the fat body were purified by CNBr-activated Sepharose 4B with anti-PGK1 monoclonal antibody. *p < 0.05, *n* = 3. **E** Interference efficiency analysis of *PGK1* at protein levels from the input of **D**. *p < 0.05, *n* = 3. **F** The Vmax of 3-PG production was determined from **A** in vitro. PGK1 proteins from the fat body were purified by CNBr-activated Sepharose 4B with the anti-PGK1 monoclonal antibody. *n* = 3. **G** and **H** Concentrations of glucose (**G**) and lactate (**H**) in *H. armigera* hemolymph from **A**. *n* = 3. **I** Phenotypes after *PGK1* knockdown as **A** (500 ng/larva administered in sixth-instar at 72 h, three times over 24 h intervals). Images were obtained after the first RNAi exposure for 80 h. **I’** Percentages of phenotypes in **I** based on three repeats (thirty larvae for each repeat). *p < 0.05, **p < 0.01, *n* = 3. **J** The pupation time from 6th-6 h to pupa 0 d after RNAi exposure in **I**. *p < 0.05, *n* = 3. **K** The average body weight of pupa after RNAi exposure in **I**. *n* = 3. Bars indicate means ± SD for three independent experiments with five larvae per replicate. Statistical analyses were performed using two-tailed Student’s *t* tests (*: *p* < 0.05 and **: *p* < 0.01)

Sixth-instar larvae at 72 h from the wandering stage were used to determine the function of PGK1 acetylation in vivo due to the high levels of acetylated-PGK1 but low levels of phosphorylated-PGK1 during metamorphosis. *PGK1* was knocked down by injecting dsPGK1 into the sixth-instar larvae at 72 h. Compared with the dsGFP injection control group, the decomposition degrees of the larval fat body were lower after dsPGK1 exposure for 60 h (Fig. 6A). Compared with the dsGFP control, immunohistochemistry showed that caspase-3 was sparingly localized in larval fat body injected with dsPGK1, suggesting that apoptosis was repressed when *PGK1* was knocked down (Fig. 6A and, A’). TEM showed that autophagosomes, autolysosomes, and apoptotic nuclei decreased in the fat body after *PGK1* knockdown in contrast to the dsGFP control (Fig. 6B, B’). Additionally, western blot demonstrated a reduction in caspase-3 cleavage and ATG8-I converting to ATG8-II in dsPGK1 compared with dsGFP (Fig. 6C and C’). Acetylated-PGK1 from the fat body decreased after dsPGK1 exposure for 60 h, compared with the dsGFP control (Fig. 6D and D’) when *PGK1* was confirmed to be knocked down by western blot (Fig. 6E). Vmax of 3-PG production of PGK1 from the fat body, as well as the hemolymph glucose and lactate concentrations, exhibited no difference between dsPGK1 and dsGFP exposure for 60 h (Fig. 6F–H), confirming that phosphorylated-PGK1 rather than acetylated-PGK1 dominated the PGK1-catalyzed glycolysis reaction. Meanwhile, statistical analysis of dsPGK1 exposure for 80 h revealed that 27% of larvae exhibited significantly delayed pupation (Fig. 6I, I’), with pupation time delayed by about 22 h on average compared with the control group injected with dsGFP (Fig. 6J). However, pupa weight did not differ between dsPGK1 and dsGFP (Fig. 6K). These data suggested that acetylated-PGK1 is essential for autophagy and apoptosis during metamorphosis.

## Discussion

**Fig. 7:**
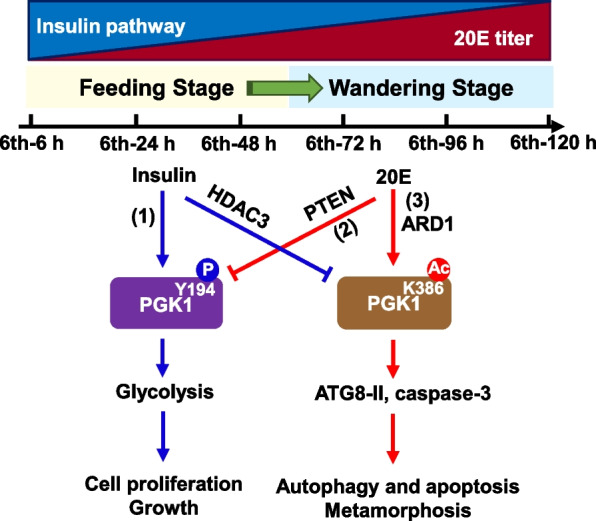
Model of proposed crosstalk between 20E and insulin signaling during metamorphosis in regulating glycometabolism and tissue remodeling. Insulin promotes PGK1 phosphorylation at Y194 to increase glycolysis and cell proliferation (1). 20E represses the expression of PGK1 and dephosphorylates PGK1 via PTEN to suppress glycolysis and cell proliferation (2). 20E induces PGK1 acetylation via ARD1 at K386 to trigger PCD, and insulin stimulates PGK1 deacetylation via HDAC3 (3). High concentrations of 20E antagonize the insulin pathway by switching the phosphorylation of PGK1 to acetylation, thus suppressing glycolysis and inducing autophagy during metamorphosis

PGK1 plays a critical role in maintaining energy supply through glycolysis and its posttranslational regulation is also responsible for cellular proliferation, autophagy, and apoptosis. Although some studies have revealed relationships between the posttranslational modification of PGK1 and its functions, little is known about the upstream regulatory mechanisms and their connection to the development of organisms. In this study, insulin promotes the phosphorylation of PGK1 during larval feeding and growth stages when 20E levels are low, thereby leading to an active role of PGK1 in glycolysis and cell proliferation. In contrast, the elevated 20E during metamorphosis promotes dephosphorylation of PGK1, thereby reducing glycolysis and elevating hemolymph glucose levels. In addition, high concentrations of 20E promote the acetylation of PGK1 to initiate larval tissue autophagy and apoptosis. We demonstrated for the first time that insulin and the steroid hormone 20E counteractively regulate PGK1 phosphorylation and acetylation to regulate glycolysis, cell growth, or PCD (Fig. 7). These findings reveal a previously underappreciated glycolytic enzyme dimension to metamorphosis.

### Insulin and 20E counteractively regulate glycolysis by altering PGK1 phosphorylation

Glucose levels are controlled by glycolysis in vivo. Glycolysis results in the breakdown of glucose, which produces pyruvate and lactate. PGK1 is one of the enzymes that catalyzes a reversible reaction in glycolysis. Insulin promotes glucose metabolism via glycolysis to produce energy [7, 30] and accumulates nutrients for cellular proliferation and body growth [31–33]. 20E arrests larval feeding and promotes pupation via binding to dopamine/ecdysteroid receptor (DopEcR) [25]. When nutrient availability is insufficient, animals obtain energy substrates through autophagy [34], generating glucose via gluconeogenesis [35]. Glucose increases during *H. armigera* metamorphosis, suggesting the repression of glycolysis and increased gluconeogenesis activity [5], although the mechanism of this change remains unclear. Here, we found that 20E represses PGK1 function in glycolysis by promoting dephosphorylation of PGK1. Moreover, knockdown of *PGK1* reduced PGK1-mediated glycolysis and larval viability at the feeding stage compared to normal larvae, thus resulting in delayed pupation and reduced pupal weight, indicating that glycolysis is crucial for larval growth and weight gain at the feeding stage. However, at the wandering stage, the larvae reached critical weight and stopped feeding; therefore, *PGK1* knockdown at the wandering stage did not affect pupal weight but affected PCD. These results suggest the complexity of PGK1 function in insect development.

PGK1 regulation has been extensively studied, with many studies concentrated on transcriptional regulation. *PGK1* can be regulated by diverse carbon sources, with glucose induction and pyruvate suppression in yeast cells [36–38]. Oxidants induce PGK1 expression in cultured human colon carcinoma cells [39] and hepatoblastoma cells [40, 41]. 20E is produced in insects and plants [42] and represents a candidate for antitumor therapy [43]. Here, we found that 20E represses PGK1 expression. PGK1-catalyzed reaction toward glycolysis was positively correlated with PGK1 phosphorylation, which is higher during feeding and growth stages, lower during molting and metamorphosis stages, and negatively correlated with 20E titers [25]. These observations demonstrate that posttranslational regulation of organismal PGK1 is temporally coupled with developmental transitions.

PGK1 T243 phosphorylation induced by interleukin-6 (IL-6) secreted from polarized M2 macrophages induces tumor cell glycolysis and tumorigenesis in human glioblastoma multiforme [44]. The epidermal growth factor receptor (EGFR) activates casein kinase 2a (CK2a) to phosphorylate PGK1 S256, leading to PGK1 interaction with cell division cycle 7 (CDC7) to accelerate DNA replication [45]. In this study, we found that PGK1 phosphorylation at Y194 promotes glycolysis and cell proliferation. In tumor cells, the decreased protein phosphatase activity of PTEN promotes PGK1 Y324 autophosphorylation, glycolysis, and cellular proliferation [46]. 20E upregulates PTEN and forkhead box protein O (FoxO) expression, inhibiting FoxO phosphorylation and stimulating FoxO nuclear localization, thereby antagonizing insulin activity in *H. armigera* [29]. This work observed that insulin induces PGK1 phosphorylation during the larval feeding stages to accelerate 3-PG production in glycolysis. However, 20E induces dephosphorylation of PGK1 via PTEN, thereby repressing PGK1-catalyzed glycolysis reaction during non-feeding metamorphosis. The high levels of 20E [25] but low expression of the insulin receptor (INR) during metamorphosis of *H. armigera* [5] result in the inhibition of insulin signaling during metamorphosis. Furthermore, the elevated 20E titers upregulate the expression of PTEN [29], which in turn dephosphorylates PGK1, thus inhibiting the PGK1-catalyzed glycolysis reaction and accumulating glucose during metamorphosis. We identified the mechanism by which 20E induces PGK1 dephosphorylation to repress glycolysis during metamorphosis.

### Insulin and 20E counteractively regulate PCD by altering PGK1 acetylation

Protein acetylation is an evolutionarily conserved post-translational modification that regulates nuclear transcription and cytoplasmic metabolism [47, 48]. Multiple lysine residues of PGK1 have been reported to be acetylated. Glutamine deprivation and hypoxia induce ARD1-dependent PGK1 K388 acetylation and promote the activation of the VPS34-Beclin1 complex, leading to the initiation of autophagy and brain tumorigenesis [23]. However, the mechanism by which PGK1 functions in autophagy and apoptosis during insect metamorphosis and its upstream signaling remains unclear.

KAT9-mediated PGK1 K220 acetylation inhibits PGK1 glycolytic activity by disrupting binding with ADP, and HDAC3-mediated PGK1 K220 deacetylation stimulates PGK1 glycolytic activity upon insulin stimulation [18]. P300/cyclic adenosine monophosphate responsive factor (PCAF) acetylated PGK1 K323 to promote its glycolytic enzymatic activity and liver cancer cell metabolism, while Sirtuin 7 deacetylated PGK1 K323 to repress its glycolytic enzymatic activity [49]. 20E counteracts insulin to trigger autophagy-related gene (ATG) expression and induce autophagy during larval-pupal metamorphosis in *Bombyx* [14]. Macroautophagy/autophagy determines apoptosis by increasing Ca^2+^ influxes due to 20E regulation in the midgut of *H. armigera* [50]. This work also found that 20E promotes the acetylation of PGK1 via ARD1, whereas insulin promotes the deacetylation of PGK1 via HDAC3. Insulin/IGF-1 signaling (IIS) [7, 51] and juvenile hormone (JH) signaling are major regulators of insect larval growth. 20E represses gene expression or induces protein modification dependent on the increased titer during metamorphosis, which must overcome the effects of IIS and JH. 20E titer decreases at the late pupal stages [25, 52]. Preadults of *H. armigera* [1] and *Drosophila* [53, 54] also exhibit increased glycolysis genes. Thus, pY-PGK1 levels elevate and Ac-PGK1 levels decline near adulthood based on the increasing ILP level [5] and decreasing 20E titer. The data indicate that insulin and 20E counteractively regulate PGK1 phosphorylation and acetylation to determine cell proliferation and PCD. PGK1 K220 acetylation in HEK293T cells represses PGK1 glycolytic activity by disrupting binding with ADP [23]. However, PGK1 is not acetylated at K218 which is conservative with PGK1 K220 of *H. sapiens* in HaEpi cells incubated with 20E. Although PGK1 K386 acetylation induced by 20E promotes PCD, it is not required for PGK1 to function in glycolysis because 3-PG production is not altered between PGK1 WT and PGK1 K386R.

PGK1 is highly acetylated during the wandering stage and plays a key role in the initiation of metamorphosis. Consequently, the wandering stage is currently used to investigate the role of PGK1 acetylation in insect development. The defective phenotypes of fat body decomposition, autophagy, and apoptosis caused by *PGK1* knockdown in the wandering stage are largely due to the reduction of acetylated-PGK1. However, only 27% of larvae exhibited delayed pupation after *PGK1* knockdown. One possible reason is that autophagy affects tissue remodeling but does not affect pupation time. To confirm the function of acetylated-PGK1, either mutation of the PGK1 acetylation site or topical inhibitor targeting PGK1 acetylation will be the most effective strategy in this non-model organism when the techniques are available.

### Different PTMs are responsible for modulating PGK1 functions under physiological conditions

*H. armigera* PGK1 shares 78.31% identity with *Drosophila* PGK1 and 75.24% identity with human PGK1 in amino acid sequence. The identification of PGK1 Y194 phosphorylation as a positive regulator for PGK1 glycolytic activity may be a broadly-distributed regulatory mechanism considering that the novel phosphorylation site is conserved from insects to humans (Additional file 1: Fig. S2B), although it differs from previously reported regulatory sites [24, 44–46]. Interestingly, the acetylation sites around K386 of *H. armigera* PGK1 are also predicted to be present in other species of insects and mammals, including around K388 in humans which functions as a protein kinase phosphorylating Beclin1 to initiate autophagy [23], suggesting conservation of acetylation in this protein region (Additional file 1: Fig. S2B). Based on the fact that autophagy is very conserved in eukaryotic cells, PGK1 acetylation-induced autophagy during insect metamorphosis may also activate the same autophagy pathway, which needs further research. These results indicate that PGK1 functions differently under different physiological conditions at different PTM sites. However, whether the PGK1 PTMs involved in this study have similar or different functions in other species needs to be confirmed by further studies.

## Conclusions

This study demonstrated that different PGK1 posttranslational mechanisms have different functions. The Y194 phosphorylated PGK1 functions as a glycolytic enzyme at the growth and feeding stages to promote cell proliferation and growth. The K386 acetylated PGK1 functions as a protein kinase at metamorphic stages to initiate autophagy and apoptosis. The phosphorylation and acetylation of PGK1 are precisely and conteractively regulated by insulin and the steroid hormone 20E. Insulin promotes PGK1 phosphorylation, and 20E represses PGK1 phosphorylation via PTEN. 20E via ARD1 stimulates PGK1 acetylation, while insulin induces deacetylation of PGK1 via HDAC3. Thus, the posttranslational modification of PGK1 regulated by insulin and 20E determines growth and metamorphosis (Fig. 7).

## Methods

### Insects and HaEpi cells

Cotton bollworms (*Helicoverpa armigera*) were reared in our laboratory on an artificial diet [55], and the *H. armigera* epidermal cell line (HaEpi) [56] was maintained according to previously reported methods [25]. Individuals stop feeding and their bodies contract during the sixth instar stage at 72–120 h, which is also referred to as the prepupae stage. After this period, the insects molt to become pupae (at 120–144 h in the sixth instar). Insects were dissected on ice and the tissues were cleaned in cold phosphate-buffered saline solution, followed by storage in liquid nitrogen until subsequent analysis. The deacetylase inhibitors trichostatin A (TSA, 5 μM) and nicotinamide (NAM, 10 mM) were added to the culture medium 12 h and 4 h before cell harvesting, respectively.

### Hemolymph glucose and lactate concentration measurements

Hemolymph from larvae, pupae, and adults (100 μL) at different developmental stages was collected and incubated with 10 μM N-Phenylthiourea (Aladdin, Shanghai, China) and EDTA.2K (Solarbio, Beijing, China, Cat. G0280) to prevent blood blackening and solidification. Samples were then centrifuged at 10,000 × g for 10 min to remove insoluble materials, followed by hemolymph glucose determination using a glucose assay kit (Enzyme-linked Biotechnology Co., Ltd., Shanghai, China). Lactate measurements were conducted using a lactate assay kit following the manufacturer’s instructions (Eton Bioscience, Inc., San Diego, USA). Photometric quantification of absorbance measurements was determined by spectrophotometry (Infinite M200PRO NanoQuant, Tecan, Grödig, Austria).

### Hormonal treatment of *H. armigera* larvae

Sixth instar 6 h larvae were subjected to 20E (H5142, Sigma Aldrich, USA; 200, and 500 ng/larva) or human insulin (P3376, Beyotime Biotechnology, 1, 5, and 10 μg/larva) injection with an equivalent amount of dimethyl sulfoxide (DMSO) or PBS injected for controls. Five microliters of solution were injected into the hemolymph of the sixth instar 6 h larvae, individually. The larvae were sacrificed at 1, 3, and 6 h after injection of different concentrations of 20E or insulin. Tissues were dissected from the sacrificed larvae for qRT-PCR (Quantitative real-time PCR) analysis or western blot. Five animals were used for per replicate and more than three biological experiments were conducted for each group.

### qRT-PCR

Total RNA was extracted using the TRIzol reagent (Tiangen Biotech, Beijing, China), and 1 μg of each RNA sample was extracted to generate cDNA for qRT-PCR. qRT-PCR reactions were performed as previously described [25]. The mRNA levels of each gene were calculated with the 2^−ΔΔCt^ method and normalized to the abundance of the housekeeping gene *β-actin* (XM_021337112.1). The relative mRNA levels for each gene are shown as fold-level changes relative to *β-actin* expression levels. Primer sequences used for qRT-PCRs are shown in Table S1 (Additional file 1).

### Antibodies

Antibodies were commercially purchased including mouse monoclonal antibody anti-PGK1 (Santa Cruz Biotechnology, Cat. sc-130335; RRID: AB_627677), mouse monoclonal antibody anti-phosphate-tyrosine (anti-pY; Santa Cruz Biotechnology, Cat. sc-7020; RRID: AB_628123), rabbit polyclonal antibodies anti-acetylated lysine (anti-Ac; Cell Signaling Technology, Cat. 9441), rabbit monoclonal antibody anti-β-actin (ABclonal, Cat. AC026), mouse anti-GFP-Tag mAb (ABclonal, Cat. AE012), mouse anti-His-Tag mAb (ABclonal, Cat. AE003), mouse control IgG (ABclonal, Cat. AC011), and alkaline phosphatase-labeled goat anti-rabbit or horse anti-mouse IgG secondary antibodies (ZSGB-BIO, Cat. ZB-2308; ZB-2310). ATG8 and Caspase3 were detected with rabbit polyclonal antibodies against *H. armigera* ATG8 and Caspase3 that were prepared in our laboratory.

### PGK1 enzyme activity measurements

PGK1 WT and PGK1 variants were overexpressed in HaEpi cells individually and PGK1-His were pulled down for the PGK1-catalyzed reaction to investigate the directionality of the PGK1 reaction. PGK1 activity was measured at room temperature in kinetic mode for 5 min by coupling with GAPDH. The absorbance at 340 nm was measured based on changes in NADH concentrations with a BioTek Synergy Neo Multi-Mode Plate Reader (BioTek, USA). PGK1 was isolated from *H. armigera* cell lysates or fat body and subjected to a PGK1-catalyzed reaction of 3-PG production assays in a reaction buffer containing 5 mM KH_2_PO_4_ (pH 7.0), 1 mM glyceraldehyde 3-phosphate (3-GAP), 0.3 mM β-nicotinamide adenine dinucleotide (β-NAD), 0.2 mM ADP, 5 mM MgSO_4_, 100 mM glycine, and 5 ng/mL GAPDH.

### Protein expression and purification

*H. armigera* tissues were extracted using an extraction buffer [40 mM Tris–HCl, 1 mM phenylmethanesulfonyl fluoride (PMSF), pH 7.5]. After completely grinding the tissues, homogenates were centrifuged at 10,000 g for 10 min at 4 °C, followed by collecting the supernatants. HaEpi cells were then resuspended in lysis buffer [25 mM HEPES (pH 8.0), 150 mM NaCl, protease inhibitors (Roche)] and the homogenate was centrifuged at 10,000 g for 10 min. Cell lysates and supernatants were purified on Ni–NTA affinity columns (GE Healthcare, Pittsburgh, USA). After washing with a 20 mM imidazole-containing buffer [25 mM HEPES (pH 8.0), 150 mM NaCl and 250 mM imidazole], fusion proteins were eluted. Protein isolation from fat body was conducted with antibody-bound-CNBr-activated Sepharose 4B. Anti-PGK1 monoclonal antibody was conjugated with CNBr-activated Sepharose 4B. Proteins were then extracted and added to the treated CNBr-activated Sepharose 4B column and incubated at 4 °C overnight with gentle shaking. The column was then washed with TBS buffer (0.1 M Tris–HCl, 0.5 M NaCl, pH 8.0), followed by elution with 200 μL of 0.1 M glycine (pH 2.5) and 10 μL of 1 M Tris–HCl (pH 8.0). Extracted crude cell lysates (50 μL) were used as input. The protease, phosphatase, and deacetylase inhibitor, trichostatin A (TSA), were added during protein extraction. The extracted and isolated proteins were then assessed by SDS-PAGE for further analysis. Protein concentrations were subsequently determined using a Micro-Bicinchoninic acid (BCA) assay kit (Pierce, Rockford, IL, USA) according to the manufacturer’s instructions. An appropriate amount of loading buffer was added to the lysates and samples were boiled for 10 min for western blot analysis. Each sample included at least three individuals and three biological replicates were used for each assay.

### Western blot

SDS-PAGE analysis was conducted using 50 μg of protein for each sample. Briefly, supernatant aliquots were loaded onto 12.5% SDS-PAGE gels. Protein ladder markers (Thermo Fisher Scientific, California, USA) were then used to identify the molecular weights of target proteins. Following electrophoresis, proteins were transferred to nitrocellulose membranes (Millipore, Darmstadt, Germany) and blocked with 5% fat-free powdered milk in Tris-buffered saline (TBS, 10 mM Tris–HCl, 150 mM NaCl, pH 7.5) at room temperature for 1 h. Blots were incubated with the primary antibody diluted to 1:2,500 with 2% BSA in TBS containing 0.1% Tween-20 (TBST), with incubation overnight at 4 °C. The membranes were washed in triplicate with TBST for 10 min each wash and blots were incubated with alkaline phosphatase (AP) conjugated secondary antibody diluted to 1:10,000 with 1% BSA in TBS-T for 1 h at room temperature. After additional washing, immunoreactivity was measured using 10 mL of 1 × TBS combined with 40 μL of 0.75% p-nitro blue tetrazolium chloride (NBT) and 30 μL of 0.5% 5-bromo-4-chloro-3-indolyl phosphate (BCIP, Sangon, Shanghai, China) in the dark, followed by scanning using a 5200 Chemiluminescence Imaging System (Tanon Science & Technology, Shanghai, China).

### Plasmids

cDNA encoding full-length *H. armigera* *PGK1* and *PTEN* were cloned into GFP or His-tagged vector (pIEX-GFP-His or pIEX-His, respectively). Expression constructs were subsequently validated by DNA sequencing. Plasmid transfection was conducted with the Quick Shuttle-enhanced transfection reagent (Biodragon-Immunotech, Beijing, China).

### Immunoprecipitation (IP)

Selected plasmids were co-transfected into HaEpi cells, followed by 20E or insulin treatment for 3 h, or exposure to DMSO or PBS for controls. Cells or tissues were lysed with lysate buffer (P0013, Beyotime Biotechnology, Shanghai, China) containing a protease inhibitor and the supernatant was collected by centrifugation at 12,000 × g for 10 min at 4 °C. The supernatant was then mixed with 50 μL of protein A and shaken at 4 °C for 1 h to eliminate non-specific binding, followed by harvesting with centrifugation. Crude cell lysate extracts (50 μL) before immunoprecipitation were used as inputs. Antibodies were added to cell lysates and incubated overnight at 4 °C, followed by incubation with 50 μL of protein A for 4 h at 4 °C. The complex was then washed with lysate buffer three times, followed by resuspension of protein A using 40 μL of lysate buffer. Immunoprecipitation was applied for western blot and liquid chromatography/tandem mass spectrometry (LC–MS/MS) analysis.

### PGK1 phosphorylation and acetylation analysis

The *PGK1* (XM_021332176.1) ORF was inserted into the vector pIEx-4-His. pIEx-4-PGK1-His was then overexpressed in HaEpi cells for 36 h and transfected with dsRNA for 24 h, followed by incubating with 20E or insulin. Samples were then boiled for 10 min after adding SDS-PAGE loading buffer and analyzed by western blot. PGK1-His was purified using a Ni^2+^-NTA affinity column and detected using western blot with the monoclonal antibody anti-pY (Santa Cruz Biotechnology, Cat. sc-7020; RRID: AB_628123) or anti-Ac (Cell Signaling Technology, Cat. 9441).

### LC–MS/MS assay

PGK1 protein was purified via immunoprecipitation from the fat body of larvae in the feeding stage (sixth-instar at 48 h). The eluent was subjected to 12% SDS-PAGE and stained with Coomassie Brilliant Blue R-250. The gels were destained with destaining solution (10% acetic acid, 5% ethanol, 85% water), and the differentiated protein bands were excised for identification of ATF2 phosphorylation modifications via LC–MS/MS analysis (PTM Biolab, Hangzhou, China).

### Measurement of cellular proliferation

HaEpi cells were transfected with overexpression plasmids for 48 h, followed by treatment with 5 μg/mL insulin. A 5-ethynyl-20-deoxyuridine (EdU) kit (Ribobio, Guangzhou, China) was used to detect cell proliferation according to the manufacturer’s protocols. Nuclei were then stained with DAPI (10 μg/mL) for 10 min at room temperature in the dark, followed by observation with a Laser Scan Confocal Microscope (Carl Zeiss LSM 700 model, Zeiss).

### RNA interference

Gene knockdowns were conducted using dsRNAs that were synthesized as previously described [25]. RNAi primers including the T7 promoter were designed for PCR (Additional file 1: Table S1).

For larval RNAi, dsRNA was diluted in nuclease-free, sterile PBS. Five hundred nanograms of sterile dsRNA was injected into the larval hemocoel. Each group contained 30 larvae, and the experiments were repeated three times. A dsGFP injection was used as a control. Insect morphology and behavior were observed after the first dsRNA injection.

### Tissue hematoxylin and eosin (HE) staining and immunohistochemical assay

Larval tissues were isolated and fixed with 4% paraformaldehyde in PBS by incubating at 4 °C overnight. Fixed tissues were gradually dehydrated. Tissues were then embedded in melted paraffin and sliced into 7 μm sections using a paraffin slicing machine. Sections were adhered to gelatin-coated glass slides and slides were dried at 42 °C overnight, followed by dewaxing. Sections were gradually rehydrated and digested with 20 mM proteinase K at 37 °C for 10 min. For tissue HE staining, the sections were then stained using an HE staining kit (Sangon, Shanghai, China) according to the manufacturer’s instructions. Positive staining signals were observed with an Olympus BX51 fluorescence microscope (Olympus, Shinjuku-ku, Japan). For immunohistochemical assay, the sections were blocked with 5% BSA at 37 °C for 30 min and then incubated with anti-caspase-3 antiserum (1: 200) at 4 °C overnight. The slides were washed six times and incubated with Alexa Fluor-488 goat anti-rabbit secondary antibody (Life Technologies) at 37 °C for 1 h. DAPI (1: 1000) was used for nuclei staining, followed by observation with a Laser Scan Confocal Microscope (Carl Zeiss LSM 700 model, Zeiss).

### TEM analysis

Tissues were fixed in 2.5% glutaraldehyde for 24 h at 4 °C and then pretreated based on standard TEM procedures. An H7650 transmission electron microscope (Hitachi, Tokyo, Japan) was used to observe autolysosomes and autophagosomes.

### Autophagy assay

HaEpi cells were transfected with GFP-ATG8-His constructs for 48 h and incubated with 20E for 6 h. GFP-ATG8-His puncta fluorescence was then observed with a fluorescence microscope (LSM 700, Zeiss).

### Caspase-3 activity assay

Caspase-3 activity was investigated using a NucView 488 caspase-3 immunocytochemistry assay kit. Cells were incubated with 5 μM NucView 488 caspase-3 substrate for 30 min at room temperature according to the manufacturer’s instructions (30029, Biotium, Fremont, CA, USA). Caspase-3 signals were then observed using a Laser Scan Confocal Microscope (LSM 700, Zeiss).

### Statistical analyses

qRT-PCR data were analyzed with the GraphPad Prism 8. Western blot bands were analyzed with the ImageJ software program and the acquired data were analyzed in GraphPad Prism 8. All data represent at least three biologically independent experiments and are indicated as scatters in the figures. Bars are shown as means ± SD. The statistical significance of differences between treatments was analyzed using a two-tailed Student’s *t* test for the comparison (*p < 0.05; **p < 0.01) or a one-way analysis of variance (ANOVA) for multiple comparisons using Duncan’s multiple comparison tests and a *p* = 0.05 significance threshold level.

## Supplementary Information

**Additional file 1:** **Figure S1.** The profiles of glucose and lactate concentrations in the hemolymph and *PGK1* mRNA in the fat body. **Figure S2.** Phylogenetic tree and protein sequence alignment of PGK1 from *H. armigera* and other species based on amino acid sequence. **Figure S3.** PGK1 is phosphorylated at Tyr194. **Figure S4.** The interference efficiencies of the genes were detected separately by qRT-PCR. **Figure S5.** High 20E titer induces PGK1 acetylation at K386. **Figure S6.** Fat body autophagy and apoptosis during the feeding stage and wandering stage. **Table S1.** The PCR primer sequences used in this paper.

**Additional file 2.** The individual data values for Fig. 1A-D, Fig. 2A-F, Fig. 3A-E, Fig. 4B-D, E’, F-I, Fig. 5A-D, E’, F’, G’, Fig. 6A’, B’, C’, D’, E–H, I’, J, K, Fig. S1A and B, Fig. S4A-C, Fig. S5A-E and Fig. S6B’, C’, D’.

**Additional file 3.** Original western blot data.

## Data Availability

All data are contained within the manuscript, including Supplemental Information.

## Abbreviations

20E: 20-Hydroxyecdysone
PCD: Programmed cell death
PGK1: Phosphoglycerate kinase 1
PTEN: Phosphatase and tensin homolog
HDAC: Histone deacetylase
ARD1: Acetyltransferase arrest-defective protein 1
ATP: Adenosine triphosphate
ILPs: Insulin-like peptides
1,3-BPG: 1,3-Bisphosphoglycerate
3-PG: 3-Phosphoglycerate
O-GlcNAc: O-linked N-acetylglucosamine
DMSO: Dimethyl sulfoxide
PBS: Phosphate-buffered saline
5F: Fifth-instar feeding larvae
5M: Fifth-instar molting larvae
6th-6 h to 6th-120 h: Sixth-instar larvae at the corresponding hours
P-0 d to P-8 d: 0- To 8-day-old pupae
F: Feeding
M: Molting
MM: Metamorphic molting
P: Pupae
Vmax: Maximum reaction rate
PTM: Post-translational modification
pY: Phosphated tyrosine
Ac: Acetylated lysine
LC–MS/MS: Liquid chromatography/tandem mass spectrometry
EdU: 5-Ethynyl-2′-deoxyuridine
HaEpi: *H. armigera* Epidermal cell line
CQ: Chloroquine
TEM: Transmission electron microscopy
DopEcR: Dopamine/ecdysteroid receptor
IL-6: Interleukin-6
EGFR: Epidermal growth factor receptor
CK2a: Casein kinase 2a
CDC7: Cell division cycle 7
FoxO: Forkhead box protein O
INR: Insulin receptor
PCAF: P300/cyclic adenosine monophosphate responsive factor
ATG: Autophagy-related gene
IIS: Insulin/IGF-1 signaling
JH: Juvenile hormone
TSA: Trichostatin A
NAM: Nicotinamide
qRT-PCR: Quantitative real-time PCR
3-GAP: Glyceraldehyde 3-phosphate
β-NAD: β-Nicotinamide adenine dinucleotide
