# Supplementary material for: 20-Hydroxyecdysone counteracts insulin to promote programmed cell death by modifying phosphoglycerate kinase 1
Source: BMC Biol. 2023 May 24;21:119. doi: 10.1186/s12915-023-01621-2 (PMC10210335; doi:10.1186/s12915-023-01621-2)
Supplement: Supplementary file 1 — Additional file 1: Figure S1. The profiles of glucose and lactate concentrations in the hemolymph and PGK1 mRNA in the fat body. Figure S2. Phylogenetic tree and protein sequence alignment of PGK1 from H. armigera and other species based on amino acid sequence. Figure S3. PGK1 is phosphorylated at Tyr194. Figure S4. The interference efficiencies of the genes were detected separately by qRT-PCR. Figure S5. High 20E titer induces PGK1 acetylation at K386. Figure S6. Fat body autophagy and apoptosis during the feeding stage and wandering stage. Table S1. The PCR primer sequences used in this paper. [file 12915_2023_1621_MOESM1_ESM.pdf]

Additional file 1
Supplementary Figures

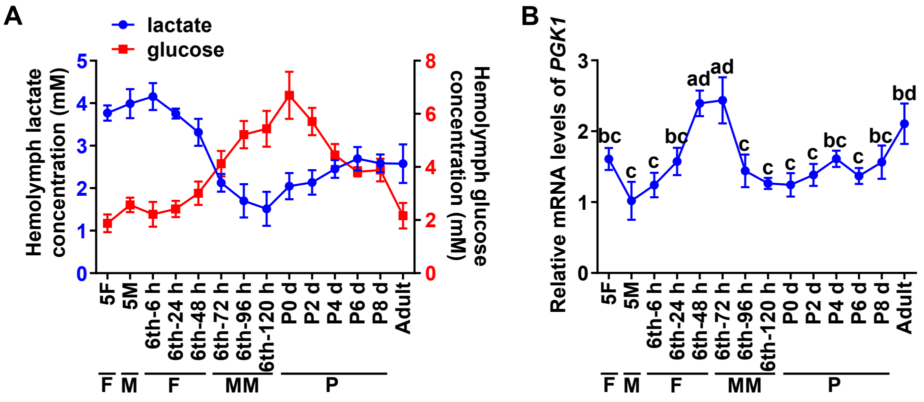

Fig. S1 The profiles of glucose and lactate concentrations in the hemolymph and *PGK1* mRNA in the fat body. **A** Glucose and lactate concentrations in *H. armigera* hemolymph from 5F to adult stages. n=3. **B** Expression profiles of *PGK1* mRNA levels in the fat body. $p < 0.05$, n=3. 5F, fifth-instar feeding larvae; 5M, fifth-instar molting larvae; 6th-6 h to 6th-120 h represent sixth-instar larvae at the corresponding hours. P-0 d to P-8 d denote 0- to 8-day-old pupae. F, feeding; M, molting; MM, metamorphic molting; P, pupae. Bars indicate means ± SD for three independent experiments with five larvae per replicate. Statistically significant differences were calculated using one-way analysis of variance (ANOVA, $p < 0.05$) tests.

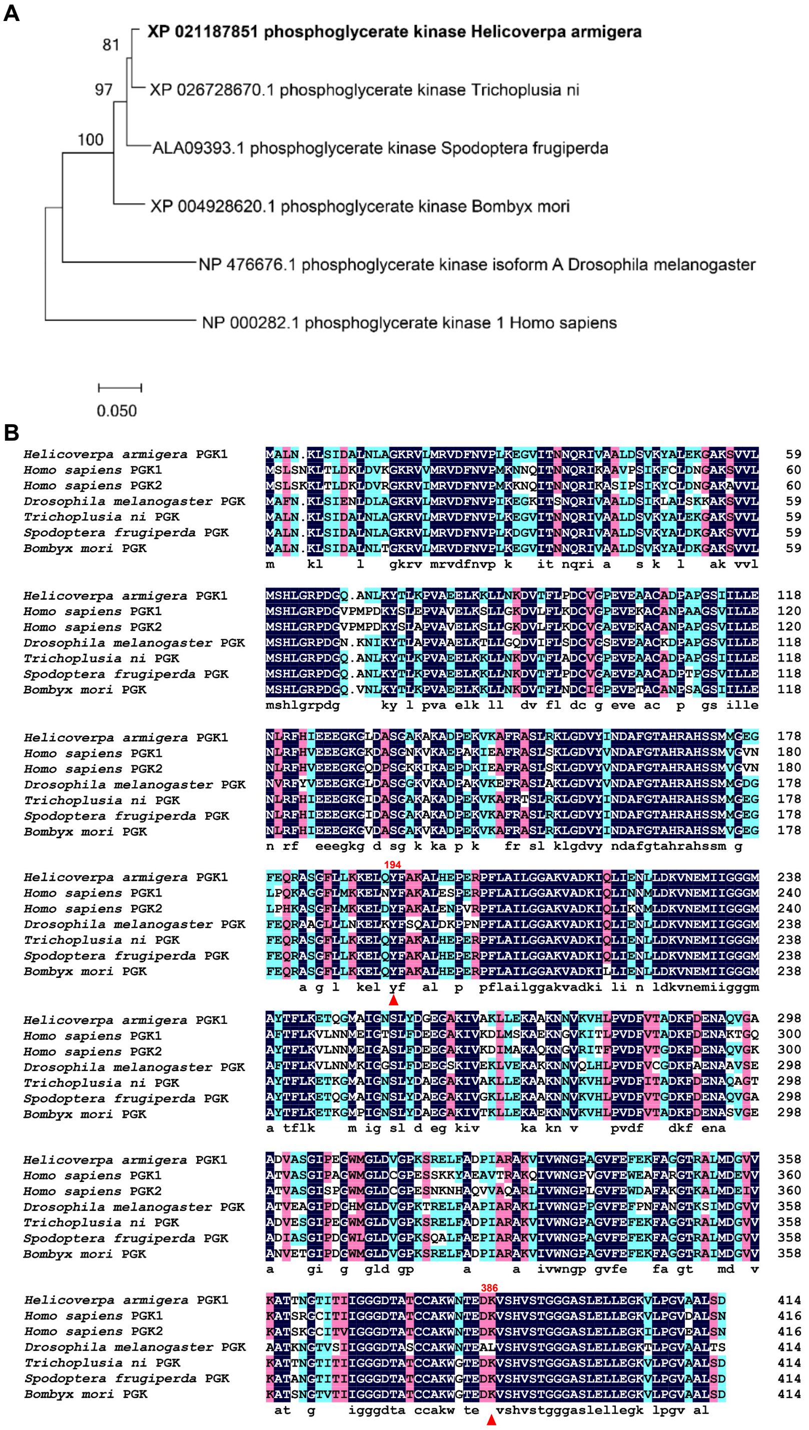

Fig. S2 Phylogenetic tree and protein sequence alignment of PGK1 from *H. armigera* and other species based on amino acid sequence. **A** Phylogenetic tree of PGK1 from *H. armigera* and other species (NJ method). Numbers above branches support values (%) based on 1,000 replicates are indicated, the scale bar represents 0.05% amino acid substitutions per site, and the GenBank accession numbers are shown before the gene names. **B** Protein sequence alignment of PGK1 from *H. sapiens* and some insects. The PTM sites in this study are marked in the red triangle.

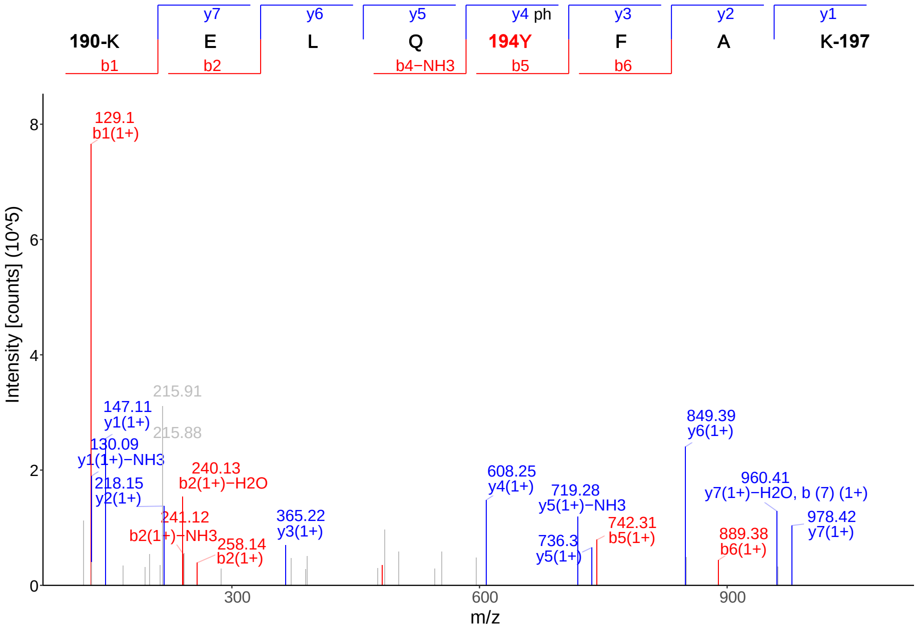

Fig. S3 PGK1 is phosphorylated at Tyr194. LC-MS/MS analysis was used to identify the phosphorylation site of PGK1 protein purified from the fat body of larvae in the feeding stage.

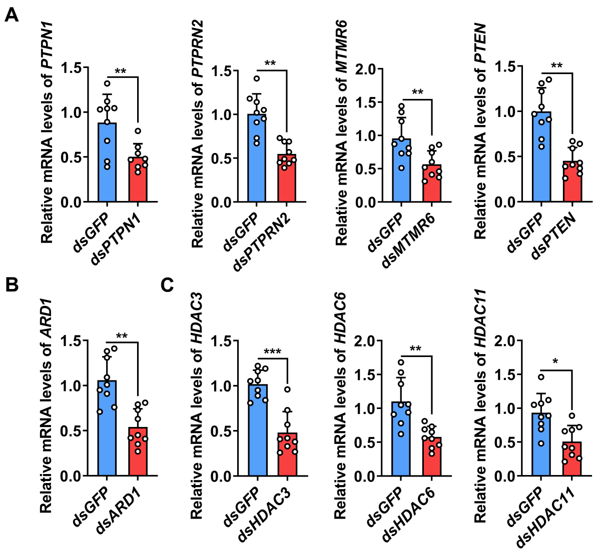

Fig. S4 The interference efficiencies of the genes were detected separately by qRT-PCR. Efficiency analysis of *PTPN1*, *PTPRN2*, *MTMR6*, and *PTEN* knockdown (**A**), *ARD1* knockdown (**B**), and *HDACs* knockdown (**C**) by qRT-PCR. *dsRNA* knockdown (500 ng/larva administered in the sixth-instar at 6 h, three times over 24 hours intervals). *dsGFP* as a control. The bars indicate mean ± SD of more than eight independent experiments with five larvae per replicate. Statistical analyses were performed using two-tailed Student's *t*-test (*: $p < 0.05$, **: $p < 0.01$, and ***: $p < 0.001$). n=8 or 9.

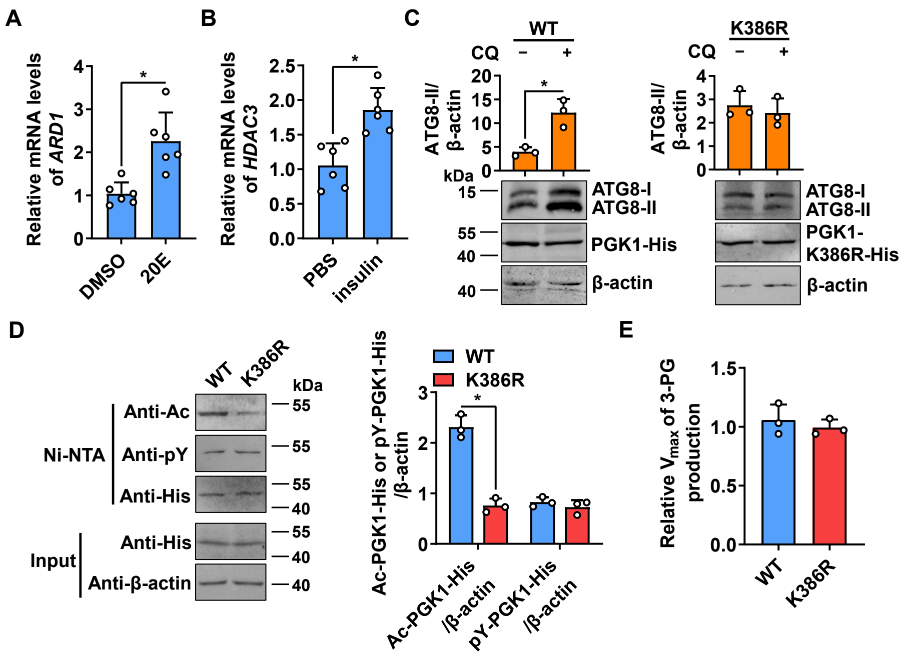

Fig. S5 High 20E titer induces PGK1 acetylation at K386. **A** 20E increased *ARD1* expression. 500 ng/larva 20E injected into the sixth-instar at 6 h for 3 hours. *$p < 0.05$, n=6. **B** Insulin increased *HDAC3* expression. 5 µg/larva insulin injected into the sixth-instar at 6 h for 3 hours. *$p < 0.05$, n=6. **C** Detecting ATG8-II levels after chloroquine (CQ) inhibits lysosomal activity. PGK1 WT-His (WT) and PGK1 K386R-His (K386R) were overexpressed in HaEpi cells for 48 hours, respectively, and then treated with 5 µM 20E for 6 hours, followed by a further 3 hours of culture with or without 50 mM chloroquine (CQ). Immunoblotting analyses were performed with the anti-ATG8 antibodies and anti-His antibody. **$p < 0.01$, n=3. **D** PGK1 phosphorylation and acetylation levels in WT and the K386R mutant. PGK1 WT-His and PGK1 K386R-His were overexpressed in HaEpi cells for 48 hours, respectively, and then treated with 5 µM 20E for 3 hours. PGK1 proteins were purified by Ni-NTA agarose beads. *$p < 0.05$, n=3. **E** PGK1 proteins were pulled down using Ni-NTA agarose beads, followed by the determination of Vmax of 3-PG production from (D). n=3. The data represent mean ± SD for more than three independent experiments with one six-well cell culture plates or five larvae per replicate. Significant differences were calculated using the two-tailed Student's *t*-test (*$p < 0.05$ and **$p < 0.01$). pY, antibody against phosphorylated tyrosine. Ac, antibodies against pan anti-acetyl lysine.

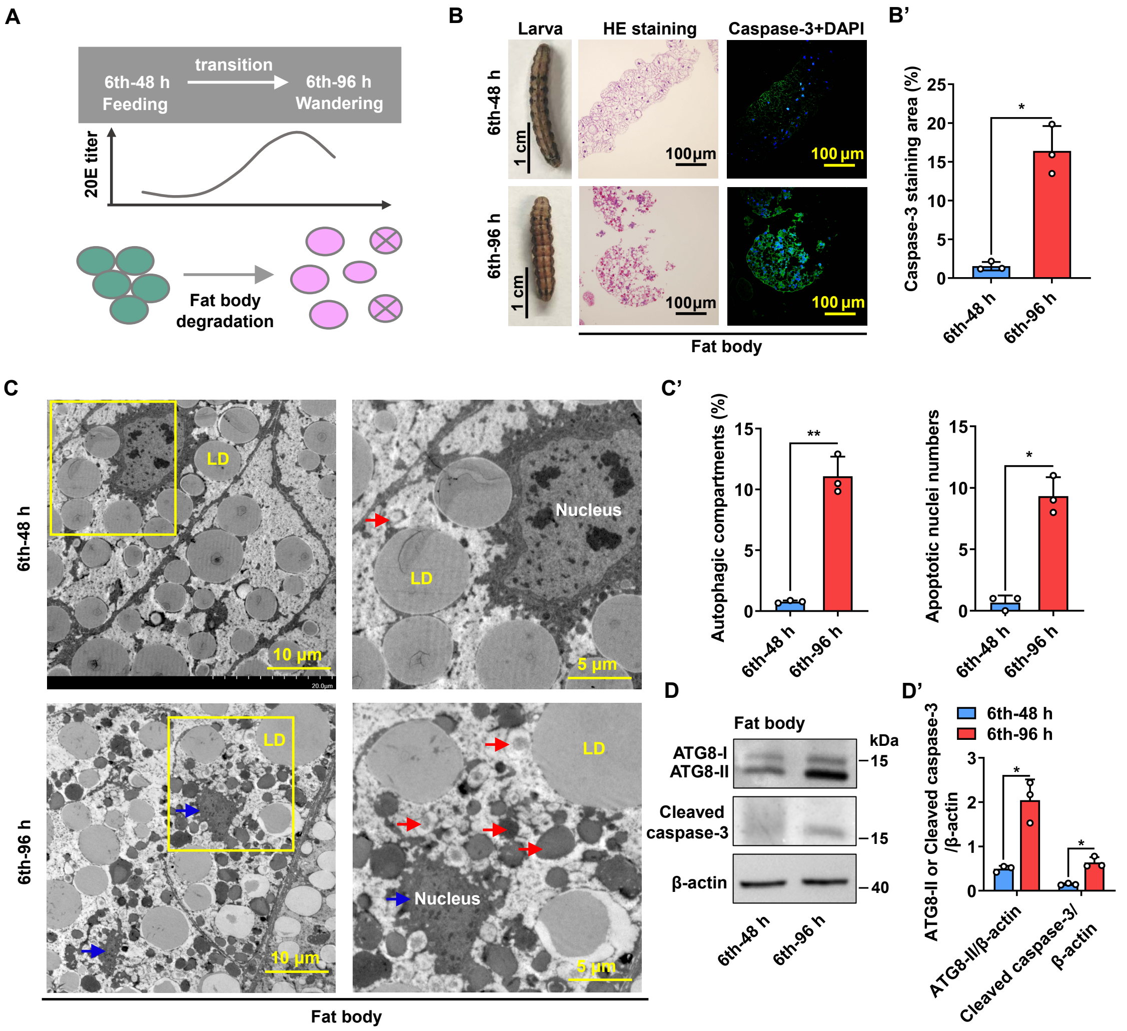

Fig. S6 Fat body autophagy and apoptosis during the feeding stage (sixth-instar at 48 h) and wandering stage (sixth-instar at 96 h). **A** Schematic of fluctuations in ecdysteroid titers and fat body degradation during the transition from the 6th-48 h of feeding stage to the 6th-96 h of wandering stage. **B** Morphology, HE staining, and caspase-3 location of the fat body from sixth-instar individuals at 48 h and 96 h. Rabbit polyclonal antibodies against caspase-3 were used as the primary antibodies. Green fluorescence indicates caspase-3 activity. Nuclei were stained with DAPI (blue). **B'** Quantification of fluorescent caspase-3 in (B). *$p < 0.05$, n=3. **C** TEM analysis of fat body from sixth-instar larvae at 48 h and 96 h. LD indicates lipid droplets. The red arrows indicate autolysosomes or autophagosomes. Blue arrows represent apoptotic nuclei. **C'** Quantification of autophagosomes, autolysosomes, and apoptotic nucleus in (C). *$p < 0.05$, **$p < 0.01$, n=3. **D** and **D'** Levels of ATG8-II and cleaved-caspase-3 in fat body from sixth-instar larvae at 48 h and 96 h. Western blot detection using anti-ATG8 and anti-caspase-3 antibodies. *$p < 0.05$, n=3. Bars indicate means ± SD for three independent biological experiments with five larvae per replicate. Asterisks indicate statistically significant differences based on two-tailed Student's *t* tests and three replicates (*: $p < 0.05$ and **: $p < 0.01$).

Supplementary Tables

Table S1. The PCR primer sequences used in this paper

Primer names	Primer sequences, 5’-3’
**RNAi**	
dsGFP-F	GCGTAATACGACTCACTATAGGTGGTCCCAATTCTCGTGGAAC
dsGFP-R	GCGTAATACGACTCACTATAGGCTTGAAGTTGACCTTGATGCC
dsRFP-F	GCGTAATACGACTCACTATAGGCTTCGCCTGGGACATCCT
dsRFP-R	GCGTAATACGACTCACTATAGGGGTGTAGTCCTCGTTGTGGG
dsPGK1-F	GCGTAATACGACTCACTATAGGGGGTGAGGGCTTTGAACAG
dsPGK1-R	GCGTAATACGACTCACTATAGGGACATCGAGACCCATCCAG
dsHDAC3-F	GCGTAATACGACTCACTATAGGGCCGACTGCCATAGTGCTACA
dsHDAC3-R	GCGTAATACGACTCACTATAGGGGTTCTCGACTGGTGGTTCCT
dsHDAC6-F	GCGTAATACGACTCACTATAGGGTGCGGCACAGACGATGAA
dsHDAC6-R	GCGTAATACGACTCACTATAGGGCGTCGTAAAGAAGCGAGTTGT
dsHDAC11-F	GCGTAATACGACTCACTATAGGGGAGCGAGGCTGGGCTATCA
dsHDAC11-R	GCGTAATACGACTCACTATAGGGCCGCCACTCGTCAGCATTA
dsARD1-F	GCGTAATACGACTCACTATAGGGAGGCCAAGTGACCTGATG
dsARD1-R	GCGTAATACGACTCACTATAGGGAAGCCAAGACTGTTGGTGTA
dsPTEN-F	GCGTAATACGACTCACTATAGGGCGGCTGACTCCAGAAATG
dsPTEN-R	GCGTAATACGACTCACTATAGGGTATCATCCAAGGCAGGTA
dsPTPN1-F	GCGTAATACGACTCACTATAGGTGGCAACCTGAATCTGTC
dsPTPN1-R	GCGTAATACGACTCACTATAGGTTCTTTCTTATCGTCCTCC
dsPTPRN2-F	GCGTAATACGACTCACTATAGGCCTGAACCAAGGGTCTACATT
dsPTPRN2-R	GCGTAATACGACTCACTATAGGCGGCTCCTATCACCAACACTAA
dsMTMR6-F	GCGTAATACGACTCACTATAGGAGCGTTCTCCCGTCTTCAC
dsMTMR6-R	GCGTAATACGACTCACTATAGGTGTTCTTATCCGCCTTCTTAC
**qRT-PCR**	
qPGK1-F	CCAGGCTCCATCATCTTGC
qPGK1-R	TAAACATCGCCCAGCTTCC
qHDAC3-F	ACGGTGTTCAGGAGGCATTCTA
qHDAC3-R	CTCCCACTTTCAGCTCCGATTT
qHDAC6-F	ACTACGCCTTACATACTCACGG
qHDAC6-R	GCGATGGGCAGGATCACT
qHDAC11-F	TCCCAAGGTAGCGGTTAT
qHDAC11-R	GCCTGCGAGTATTGAGCC
qARD1-F	TTGGGCTTGCACAGAAACTTAT
qARD1-R	GGCATCTTCTCCGTCAGCATAG
qPTEN-F	TCTTCCACTTCTGGTTCA
qPTEN-R	GTGTTTATGCTGCTTATCC
qPTPN1-F	TCAACATCCAGGAGACGCTT
qPTPN1-R	ATGACGGCTTGGTAGCAGAA
qPTPRN2-F	ACAACCCACCCCTAACACCA
qPTPRN2-R	CTTCTGGGCAAGGATTCGTT
qMTMR6-F	CAGAGAATGAATGTGCCTAACG
qMTMR6-R	CTCGCTTGGGTATGTGTCG
qβ-actin-F	CCTGGTATTGCTGACCGTATGC
qβ-actin-R	CTGTTGGAAGGTGGAGAGGGAA
**Overexpression**	
PGK1-OF	TACTCACAATTGGATGGCTTTAAATAAACTAAGT
PGK1-OR	TACTCAGGCGCGCCGATGCGTCAGACAGGGCTGCAAC
PTEN-OF	TACTCAGGATCCCATGGGTATTTGCGTGAGC
PTEN-OR	TACTCAGGTACCCAAGTATGTAGATTCACC
