# Supplementary material for: 20-Hydroxyecdysone counteracts insulin to promote programmed cell death by modifying phosphoglycerate kinase 1
Source: BMC Biol. 2023 May 24;21:119. doi: 10.1186/s12915-023-01621-2 (PMC10210335; doi:10.1186/s12915-023-01621-2)

Figure 2B

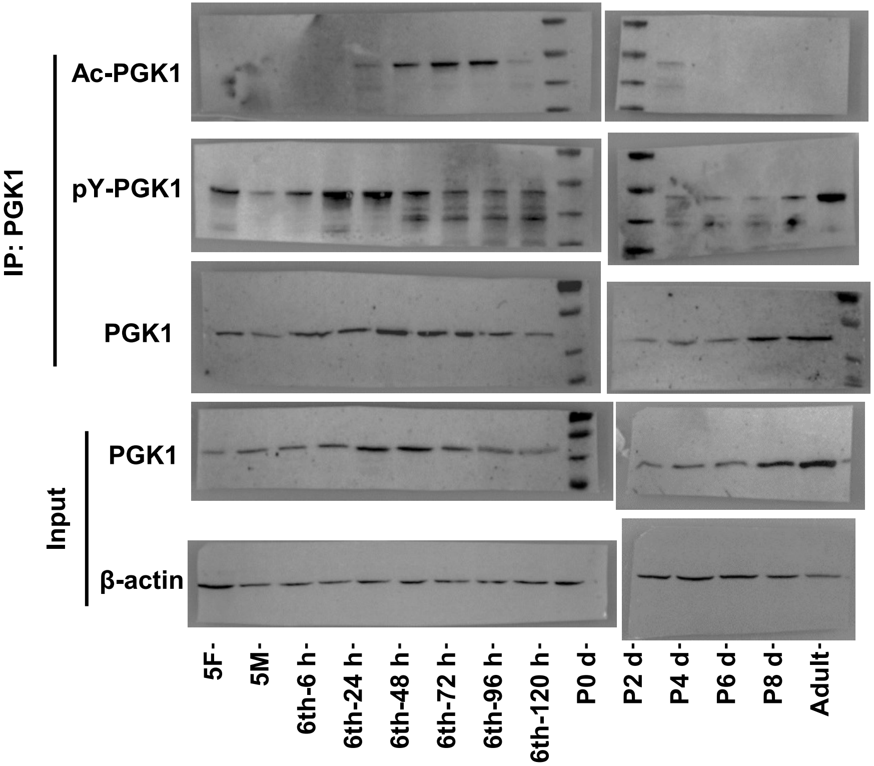

Figure 2C

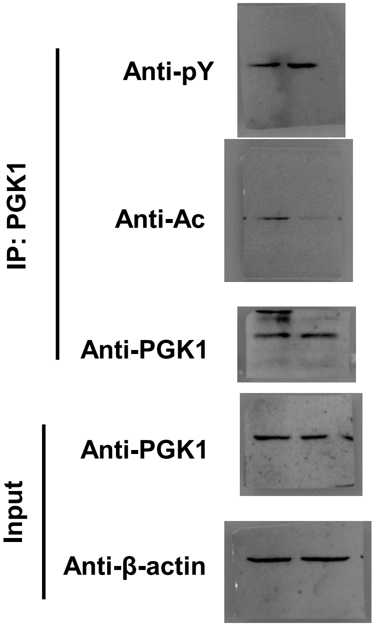

Figure 2E

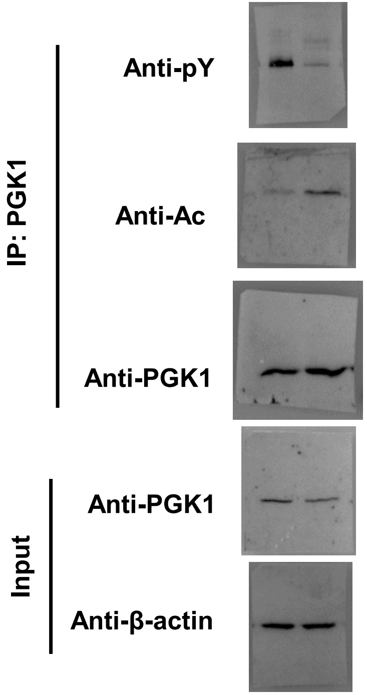

Figure 3B’

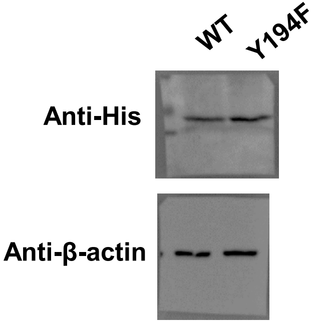

Figure 3C

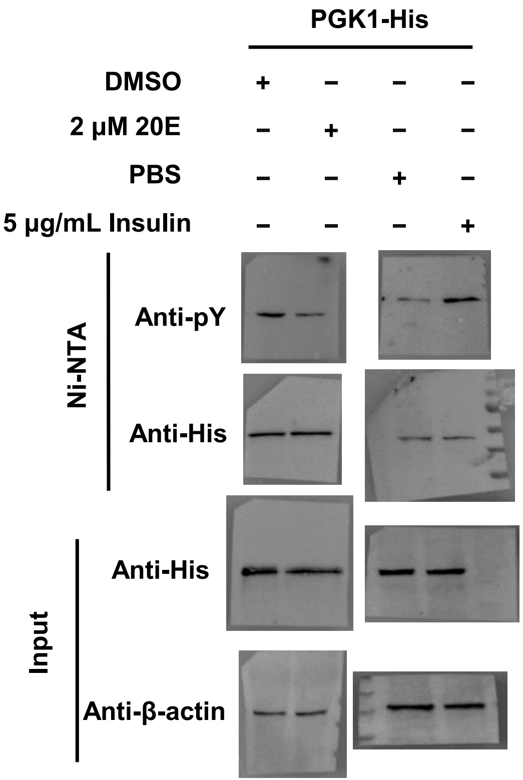

Figure 3D

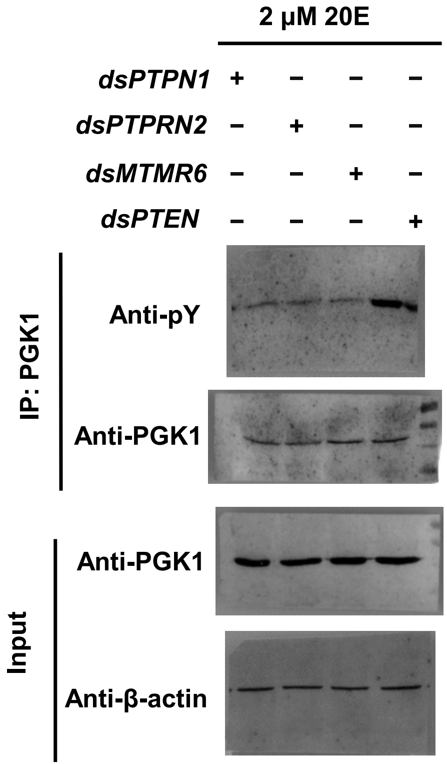

Figure 3E

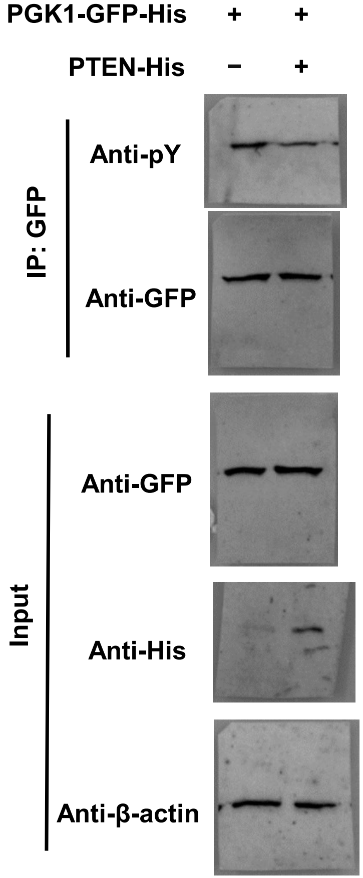

Figure 4E

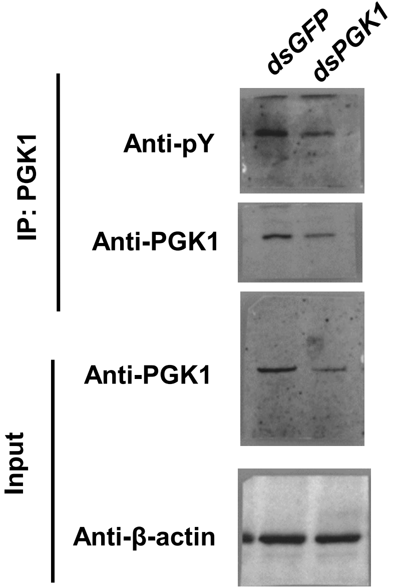

Figure 5A

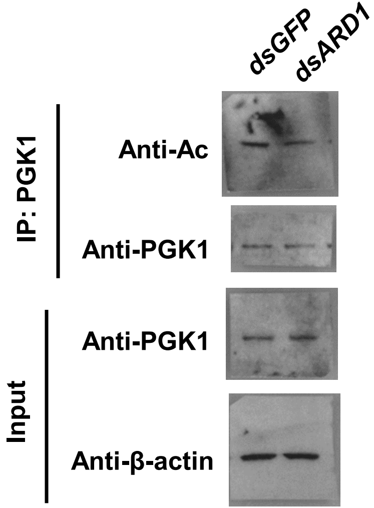

Figure 5B

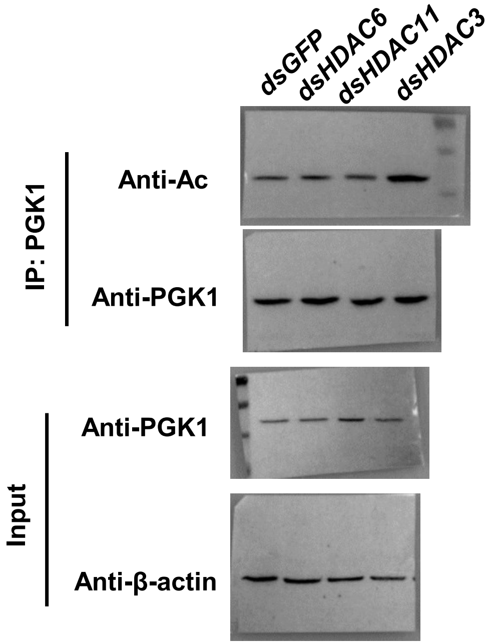

Figure 5C

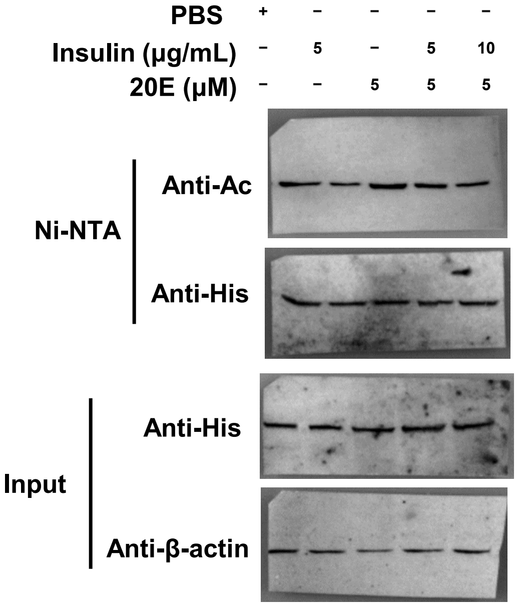

Figure 5D

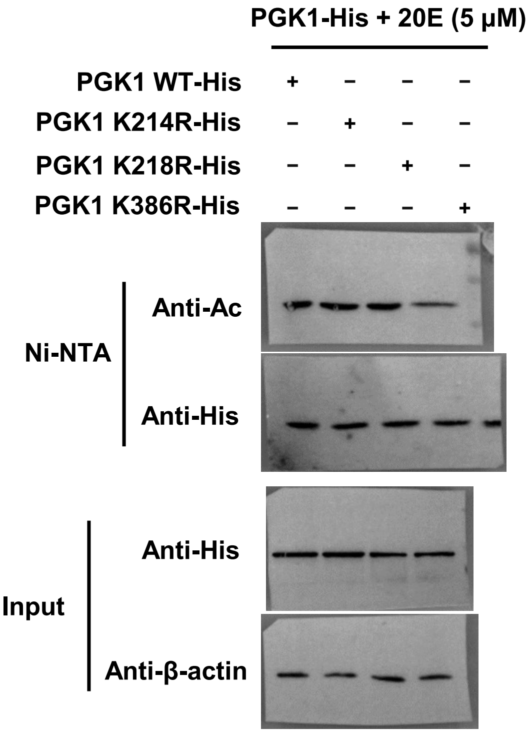

Figure 5E

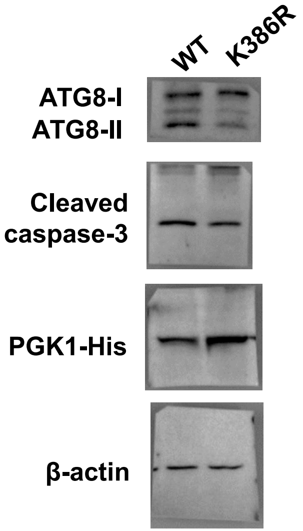

Figure S5C

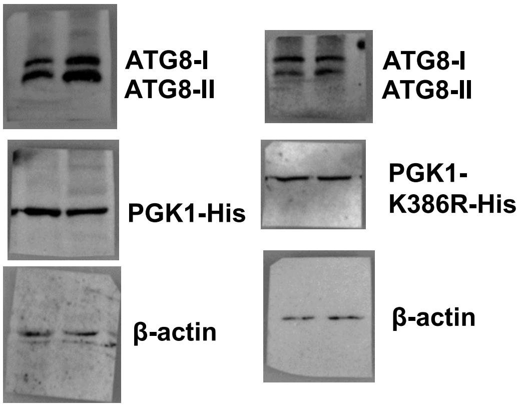

Figure S5D

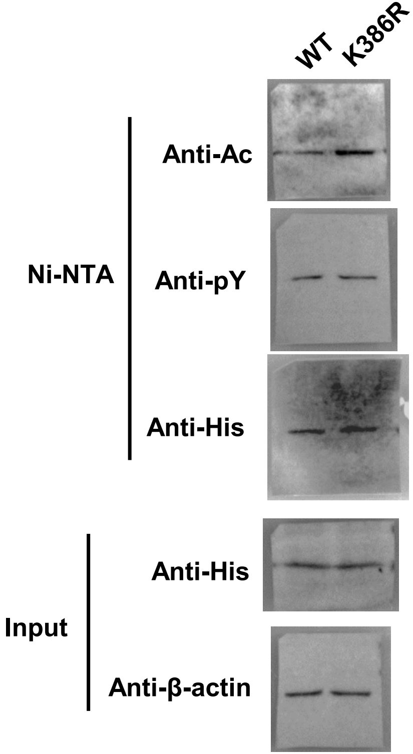

Figure S6D

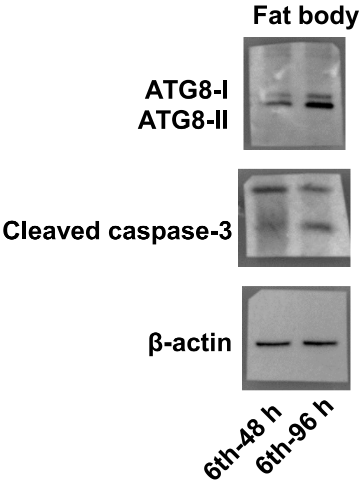

Supplement: Supplementary file 3 — Additional file 3. Original western blot data. [file 12915_2023_1621_MOESM3_ESM.pdf]
